# New resources for the Drosophila 4th chromosome: FRT101F enabled mitotic clones and *Bloom syndrome helicase* enabled meiotic recombination

**DOI:** 10.1093/g3journal/jkac019

**Published:** 2022-01-27

**Authors:** Samuel L Goldsmith, MaryJane Shimell, Petra Tauscher, Samantha M Daly, Osamu Shimmi, Michael B O’Connor, Stuart J Newfeld

**Affiliations:** 1 School of Life Sciences, Arizona State University, Tempe, AZ 85287-4501, USA; 2 Department of Genetics, Cell Biology and Development, University of Minnesota, Minneapolis, MN 55455, USA; 3 Institute of Biotechnology, University of Helsinki, Helsinki 00014, Finland; 4 Institute of Molecular and Cell Biology, University of Tartu, Tartu 51010, Estonia

**Keywords:** adult brain, dCORL/Fussel/SKOR, dILP2/dILP5, MARCM

## Abstract

Genes on the long arm of the *Drosophila melanogaster* 4th chromosome are difficult to study because the chromosome lacks mitotic and meiotic recombination. Without recombination numerous standard methods of genetic analysis are impossible. Here, we report new resources for the 4th. For mitotic recombination, we generated a chromosome with an FRT very near the centromere in 101F and a derivative that carries FRT101F with a distal ubiquitously expressed GAL80 transgene. This pair of chromosomes enables both unmarked and MARCM clones. For meiotic recombination, we demonstrate that a *Bloom syndrome helicase* and *recombination defective* double mutant genotype can create recombinant 4th chromosomes via female meiosis. All strains will be available to the community via the Bloomington Drosophila Stock Center. Additional resources for studies of the 4th are in preparation and will also be made available. The goal of the 4th Chromosome Resource Project is to accelerate the genetic analysis of protein-coding genes on the 4th, including the 44 genes with no demonstrated function. Studies of these previously inaccessible but largely conserved genes will close longstanding gaps in our knowledge of metazoan development and physiology.

## Introduction

There are 79 protein-coding genes on the long arm of the Drosophila 4th chromosome. By sequence, they belong to many multigene families and for the fraction with an existing mutation, they are often essential for survival and/or fertility. For example, 4th genes include: (1) transcription factors belonging to the forkhead, paired box (Pax), high mobility group (HMG) and zinc finger families, (2) enzymes belonging to the calcium/calmodulin-dependent, cyclin-dependent, and Tau-tubulin kinase families, (3) receptors belonging to the dopamine, plexin, and ephrin families, and (4) housekeeping genes such as kinesins, guanine nucleotide exchange factors and ATP binding proteins. Thirty-five genes on the 4th have lethal mutations and 10 have sterile mutations (6 have both). The remaining 44 genes are either homozygous viable when mutant with an unidentified adult phenotype or completely unstudied.

Fifty-nine of the 79 protein-coding genes (75%) have distinct human counterparts. Among the human homologs are conserved signaling molecules in the Hedgehog (*cubitus interruptus* and *fd102C* are GLI and FOXL1), Wingless (*pangolin* and *legless* are TCF and BCL9), and TGF-β pathways (*myoglianin* and *activin-β* are Myostatin and INHBB). There are also numerous neuronal genes: *unc-13* (UNC13A regulates α-secretase; Rossner *et al.* 2004), *sox102f* (SOX5 causes Lamb-Shaffer syndrome; Lamb *et al.* 2012), *mGluR* (GRM3 mutations may cause memory loss; de Quervain and Papassotiropoulos 2006) and *dCORL* (*fussel* in Flybase; SKOR1 is linked to ataxia; Dimitriadou *et al.* 2020).

A scan for the human homologs in 4 disease databases showed that 68% of the human genes with relatives on the 4th have a disease association. The number and diversity of unstudied genes, the conservation of a majority of them in humans and the significant fraction of human homologs with disease connections provide a strong rationale for developing new resources for the genetic analysis of mutations on the 4th.

The process of generating homozygous mutant clones employs the unnatural process of mitotic recombination. Originally stimulated by X-irradiation (Stern 1934), FLP-FRT mediated mitotic recombination significantly improved the ability of the investigator to choose the cell that would become the clone progenitor (Golic 1991). In both cases, the mutant cells in the clone were nonautonomously marked by absence of a visible phenotype present in all other cells (e.g. yellow body color or multiple wing hairs). This worked well in tissues where cells are largely uniform, the tissue is compact and cell migration is limited (e.g. appendages and abdomen).

Nonautonomous marking of mutant clones does not work well in the central nervous system where the high density and irregular shape of neurons impedes the analysis. Melding UAS-GAL4-GAL80 (a tissue-specific expression/repression scheme) with FLP-FRT allowed the release of a repressible marker exclusively in the mutant cell and its clonal descendants. This autonomous labeling method is called MARCM (Lee and Luo 1999) and it is widely used by fly neuroscience and stem cell researchers. Currently, MARCM is not feasible for studying mutants on the 4th due to the lack of mitotic recombination. In addition, on other chromosomes, the first step in the creation of a marked homozygous mutant clone is typically to recombine the mutant of interest onto the FRT chromosome. The absence of meiotic recombination on the 4th prevents this first step even after an FRT-bearing chromosome becomes available.

Beyond the lack of recombination, the highly heterochromatic nature of the 4th further complicates genetic analysis (Sun *et al.* 2004; Riddle and Elgin 2018). To overcome these obstacles we created new resources for the 4th. For mitotic recombination, we generated a 4th chromosome with an FRT very near the centromere in 101F and a derivative that carries FRT101F with a distal ubiquitously expressed GAL80 transgene. This pair of chromosomes enables both unmarked and MARCM clones. For meiotic recombination, we demonstrate that a *Bloom syndrome helicase* and *recombination defective* double mutant genotype can create recombinant 4th chromosomes via female meiosis. All strains will be available to the community via the Bloomington Drosophila Stock Center. Additional resources for studies of the 4th are in preparation and will also be made available. The goal of the 4th Chromosome Resource Project is to accelerate the genetic analysis of protein-coding genes on the 4th, including the 44 genes with no demonstrated function. Studies of these previously inaccessible but largely conserved genes will close longstanding gaps in our knowledge of metazoan development and physiology.

## Materials and methods

### Drosophila stocks

A BL prefix indicates the stock is available from the Bloomington Drosophila Stock Center.

#### Mitotic recombination resource generation

(1) *TI{TI}FRT101F-DsRed+* and *TI{FRT.Tub.GAL80.O}101F-DsRed+* construction: BL55821 *yw M{GFP[E.3xP3]=vasCas9.RFP}ZH-2A* and BL9549 *w; P{w[+mC]=ActGFP}unc-13[GJ]/In(4)ci^D^*.

(2) Testing *TI{FRT.Tub.GAL80.O}101F* suppression of dCORL.GAL4: BL5132 *w P{w[+mC]=Tub.GAL80}LL1* and BL1521 *w; P{w[+mC]=UAS.GFP.S65T}.* BL1521 was employed as part of the recombinant line *yw; P{w[+mC]=dCORL.GAL4(-).PT} P{w[+mC]=UAS.GFP.S65T}*/*SM6A* that we refer to as dCORL.GAL4. To validate dCORL.GAL4 we compared its expression to the dCORL reporter AH.lacZ (w; *P w[+mC]; HZR dCORL.5'AH*; Tran *et al.* 2018a) and BL854 *w; P{w[+mW.hs]=GawB}ey[OK107]/In(4)ci^D^.*

(3) Testing *TI{TI}FRT101F* and *TI{FRT.Tub.GAL80.O}101F* recombination: For MARCM clones the lines BL6420 *yw P{ry[+t7.2]=70FLP}3F* and dCORL.GAL4 were employed.

#### Meiotic recombination resource generation

(1) X to 4th transposition: BL30040 *yw P{y[+t7.7]=ET.GAL80}*; BL2078 *yw; H{w[+mC]=PDelta2-3}HoP2.1 CyO*/*PPO1^B^*^c^; BL50872 y*w; PBac{y[+mDint2]=HpaI-GFP.A}unc-13^YD0079^* (homozygous viable protein trap); and BL42700 *yw; lgs^17E^/In(4)ci^D^*.

(2) *Blm rec* enabled meiotic recombination: *Blm^N1^ rec^2^ P{w[+mW]=UASp.Blm}/TM6B* and *y; Blm^D2^ ry rec^1^ Ubx^bx34e^ P{w[+mW]mat-α.GAL4}/TM6B Tb^1^* (gifts of Jeff Sekelsky; Hatkevich *et al.* 2017); BL18376 *w; PBac{w[+mC]=WH}Abcd1*^f00836^; BL6782 *w; P{w[+mC]=lacW}pum^bem/^TM6* and BL9549 *w; P{w[+mC]=ActGFP}unc-13[GJ]/In(4)ci^D^* (balancer with ubiquitous GFP). 

### Genetics

#### Mitotic recombination resource generation

(1) *TI{TI}FRT101F-DsRed+* and *TI{FRT.Tub.GAL80.O}101F-DsRed+* transformation: The CRISPR Optimal Target Finder (Gratz *et al.* 2014) was employed to identify a list of candidate target sites in the most proximal band on the long arm of the 4th chromosome (101F). From the list, the region between the long noncoding RNA CR4440 (46,074 bp) and *ci* (47,710 bp) was chosen. To increase the efficiency of Cas9 cleavage, 2 targets that differed by 1 bp in their cleavage sites (46,994 bp and 46,995 bp) and that contained zero predicted off-target sites were designed. The targets with the PAM are: GCCTCCCC↓GTGTTGTCCCGT GGG and GGCCTCCC↓CGTGTTGTCCCG TGG. Sense and antisense oligonucleotides (Supplementary Table 6) for the 2 guides were cloned into pU6-BbsI-chiRNA (Addgene #45946; Gratz *et al.* 2013). Each of the guide RNAs was injected with a homology dependent repair plasmid, either *TI{TI}FRT101F-DsRed+* or *TI{FRT.Tub.GAL80.O}101F-DsRed+*, into embryos containing maternal Cas9 (BestGene, Chino Hills). G0 adults were mated to *w^1118^* flies and screened for RFP+ progeny. Subsequent mating of RFP+ flies to *P{w[+mC]=ActGFP}unc-13[GJ]/In(4)ci^D^* led to sibmating and creation of a homozygous stock. Segregation of RFP+ away from *ci^D^* was the initial confirmation that RFP+ was inserted on the 4th. Judicious use of males in these crosses also removed the maternal Cas9 transgene on the X.

(2) Removal of DsRed by intrachromosomal recombination: Five to 10 males with *TI{TI}FRT101F-DsRed+* or *TI{FRT.Tub.GAL80.O}101F-DsRed+* were crossed independently to an equal number of females that were *yw P{ry[+t7.2]=70FLP}3F; Sb/TM6B; P{w[+mC]=ActGFP}unc-13[GJ]/In(4)ci^D^*. Adults were removed after 1 day and a heat-shock/recovery cycle performed once per day for 4 days at 37^°^C starting on Day 2. Males that were *Sb* and *ci^D^* were collected and screened for DsRed fluorescent mottling in the eye indicating somatic recombination. The *P{w[+mC]=ActGFP}unc-13[GJ]* as the 4th balancer is not recommended here because *w*+ masks DsRed fluorescence. Single males with DsRed mottled eyes were crossed to females who were *w*; *P{w[+mC]=ActGFP}unc-13[GJ]/In(4)ci^D^* and the *ci^D^* male progeny screened for complete loss of DsRed in the eye indicating germline recombination. Single DsRed negative *ci^D^* males that lost *P{ry[+t7.2]=70FLP}3F* during crossing and that did not display *Sb* were again crossed to females who were *w*; *P{w[+mC]=ActGFP}unc-13[GJ]/In(4)ci^D^* to create DsRed negative females without *P{ry[+t7.2]=70FLP}3F.* Sibmating established a homozygous stock and the remaining FRT was sequenced.

#### Meiotic recombination resource generation

(1) X to 4th transposition: Females homozygous for *P{y[+t7.7]=ET.GAL80}* the on X were crossed to males bearing *w; H{w[+mC]=PΔ2–3}HoP2.1 CyO*/*PPO1^B^*^c^ an immobile P transposase source. Male progeny with *P{y[+t7.7]=ET.GAL80}* and *CyO* were collected and crossed to females heterozygous for *ci^D^*. From the progeny, 200 males that retained the paternal *P{y[+t7.7]=ET.GAL80}* indicating movement off the X chromosome and displayed the maternal *ci^D^* chromosome were crossed in single pairs to females homozygous for *PBac{y[+mDint2]=HpaI-GFP.A}unc-13^YD0079^* on the 4th (abbreviated *unc-13*:GFP). Two males with progeny that displayed 100% segregation of *P{y[+t7.7]=ET.GAL80}* away from *ci^D^* had their heterozygous *P{y[+t7.7]=ET.GAL80}*/*unc-13*:GFP siblings collected. These were sibmated and homozygous *P{y[+t7.7]=ET.GAL80}* progeny sibmated for a stock. The 2 new insertions were named *P{y[+t7.7]=ET.GAL80}MW1* and *P{y[+t7.7]=ET.GAL80}MW2.* Genomic DNA was extracted from flies of each strain. The 4th chromosome insertion sites were identified by inverse PCR and sequencing.

(2) *Blm rec* enabled meiotic recombination: Prior to starting this scheme, all stocks were given a *yw* background. The parental cross was females heterozygous on 3rd for *Blm^N1^ rec^2^ P{w[+mW]=UASp.Blm}* and homozygous for *PBac{w[+mC]-WH}Abcd1^f00836^* on 4th with males heterozygous for *Blm^D2^ ry rec^1^ Ubx^bx34e^ P{w[+mW]mat-α.GAL4}* on 3rd and homozygous for *P{y[+t7.7]=ET.GAL80}MW1* on 4th. From the progeny, *Blm rec* double mutant females capable of recombination on the 4^th^ who were also transheterozygous for *PBac{w[+mC]-WH} Abcd1^f00836^* over *P{y[+t7.7]=ET.GAL80}MW1* were collected. In the F1 cross, recombination competent females were mated to males carrying *TM6* and *ci^D^*. From the progeny, males carrying *PBac{w[+mC]-WH}Abcd1^f00836^ P{y[+t7.7]=ET.GAL80}MW1* and *ci^D^* that displayed an enhanced *Ubx* phenotype (indicating the presence of *Ubx^bx34e^*) and *TM6* were collected. This step eliminated the *Blm^N1^ rec^2^ P{w[+mW]=UASp.Blm}* chromosome. In the F2 cross, 963 of these males were individually mated with *ci^D^* heterozygous females. From the progeny, 9 candidate recombinant males with *PBac{w[+mC]-WH}Abcd1^f00836^ P{y[+t7.7]=ET.GAL80}MW1* and *ci^D^* plus a typical *TM6-Ubx* phenotype were collected. This step eliminated the *Blm^D2^ ry rec^1^ Ubx^bx34e^ P{w[+mW]mat-α.GAL4* chromosome. In the F3 cross, the 9 candidate recombinant males were individually mated to heterozygous *ci^D^* females. From the progeny of 7 surviving males, *PBac{w[+mC]-WH}Abcd1^f00836^* and *P{y[+t7.7]=ET.GAL80}MW1* over *ci^D^* candidate recombinant males and females were collected. In the F4 cross, males and females with *PBac{w[+mC]-WH}Abcd1^f00836^ and P{y[+t7.7]=ET.GAL80}MW1* over *ci^D^* from each of the 7 surviving males were sibmated. Progeny carrying *PBac{w[+mC]-WH}Abcd1^f00836^* and *P{y[+t7.7]=ET.GAL80}MW1* that were not non-*ci^D^* were again sibmated to create 7 homozygous candidate recombinant 4th chromosome stocks. In the F5 cross, *PBac{w[+mC]-WH}Abcd1^f00836^* and *P{y[+t7.7]=ET.GAL80}MW1* over *ci^D^* male progeny from F4 of each stock were crossed to *yw* females to test for 100% co-segregation of *PBac{w[+mC]-WH}Abcd1^f00836^* and *P{y[+t7.7]=ET.GAL80}MW1* away from *ci^D^*. Progeny from 3 candidates segregated perfectly.

(3) *y* wing margin bristle clones: *yw*: *PBac{w[+mC]-WH}Abcd1^f00836^* and *P{y[+t7.7]=ET.GAL80}MW1* males from each of the 3 perfectly segregating recombinant lines were crossed to *yw P{ry[+t7.2]=70FLP}3F*; *PBac{w[+mC]-WH}Abcd1^f00836^* females. After 2 days, the parents and progeny were subject to a 1-h heat-shock at 37°C. Parents were removed after the third day and the vials subject to 1-h heat-shocks on Days 4 and 6. 200 adult wings were mounted and *y* margin bristle clones scored as described (Quijano *et al.* 2011).

### Molecular biology

#### Mitotic recombination resource generation

(1) *TI{TI}FRT101F-DsRed+* and *TI{FRT.Tub.GAL80.O}101F-DsRed+* construction: Details including plasmid maps and oligonucleotide sequences are in Supplementary Figs. 1–4 and Supplementary Tables 1–8. In brief, the NEBuilder Assembly Tool (https://nebuilder.neb.com/#!/) was utilized to design oligonucleotides for Gibson (2011) assembly of 3 plasmids that were then utilized to generate transformant lines. For assembly, PCR fragments were amplified using NEB Phusion High-Fidelity DNA polymerase (#M0530), and synthetic DNA fragments were created employing GenScript. Final assembly employed NEBuilder HiFi DNA Assembly Master Mix (#E2621). The first plasmid *TI{TI}FRT101F-DsRed+*has an attP site proximal to the FRT sites. This attP has not been tested for integration of attB plasmids and would be unaffected by recombination at the FRT sites*.* The second plasmid has an attP site distal to the FRT sites. This attP was refractory to the integration of numerous attB plasmids and the stock has not been donated. The plasmid however was a valuable intermediate in the construction of the *TI{FRT.Tub.GAL80.O}101F-DsRed+* plasmid.

(2) Sequence verification: Candidates from *TI{TI}FRT101F-DsRed+* injection were fully sequenced prior to FLP out of the DsRed gene (Supplementary Table 4, rows 1/3 and 4/5 plus Supplementary Table 7 all rows). After FLP-out of DsRed, the remaining FRT site was re-verified (Supplementary Table 4, rows 1/4 plus Supplementary Table 7 rows 1/2). Candidates from the *TI{FRT.Tub.GAL80.O}101F-DsRed+* injection had the arm1-FRT-Tub junction and the GAL80-arm2 junction sequenced (Supplementary Table 4, rows 2/6 plus Supplementary Table 8, rows 1/2). After FLP-out of DsRed, the FRT site was re-verified (Supplementary Table 4, rows 1/4)*.* PCR primers from genome sequences beyond arm1 and arm2 were employed to confirm integration of *TI{TI}FRT101F* and *TI{FRT.Tub.GAL80.O}101F* at the desired location after DsRed removal (Supplementary Table 8, rows 3–5).

(3) Genomic DNA and PCR: Genomic DNA was extracted from 1 or 2 adult flies (Gloor *et al.* 1993). Two microliters of extract was PCR amplified for a region of interest (Expand High Fidelity PCR System, Roche) and then sequenced with either the PCR primers or an internal primer (Supplementary Tables 4, 7, and 8). All PCR reactions for the 4th were done in 4 mM MgCl2.

(4) *P{w[+mC]=dCORL.GAL4(-).PT}* construction: Details including chromosome and plasmid maps are in Supplementary Figs. 5–7. In brief, the entire 16,954bp intergenic region between the *dCORL* and *toy* coding exons was cloned into pPTGAL (Sharma *et al.* 2002). The proximal end is the *SmaI* site in *dCORL* intron1 and includes the long noncoding RNA *sphinx*. The distal end is the *BamHI* site in *twin of eyeless* (*toy)* intron1, the adjacent divergently transcribed gene. As enhancers can operate in both directions and the region has been shown to contain multiple intertwined, tissue-specific enhancers and repressors (Tran *et al.* 2018a) the region was cloned in both directions (forward and reverse). Forward aligns with the genomic organization (GAL4 next to *toy*). In the reverse direction, GAL4 is next to *dCORL*. Forward transgenes had no expression while reverse transgenes generated expression that mimicked *dCORL* in dILP2 expressing cells of the adult brain. Transformant strain A carrying *P{w[+mC]=dCORL.GAL4(-).PT}* on the 2nd was recombined with a 2nd chromosome carrying *P{w[+mC]=UAS.GFP.S65T}*. We refer to the recombinant 2nd chromosome as dCORL.GAL4.

#### Meiotic recombination resource generation

Inverse PCR: *P{y[+t7.7]=ET.GAL80* was derived from PLacW via PGALW and thus we followed the standard BDGP protocol (https://www.fruitfly.org/about/methods/inverse.pcr.html) for inverse PCR. Briefly, genomic DNA from *P{y[+t7.7]=ET.GAL80}*MW1 and *P{y[+t7.7]=ET.GAL80}*MW2 homozygous flies was digested with *HinP1I*. Inverse PCR primers for the 5' end were PLac4 and PLac1 and primers for the 3' end were Pry4 and PLw3-1.

### Immunohistochemistry

#### Mitotic recombination resource generation

(1) dCORL.GAL4 expression: one-day-old female adults were stained for GFP, FasII, and dILP2 or lacZ as described (Tran *et al.* 2018b). *sphinx* transcript expression was as described in Supplementary Fig. 8.

(2) Testing *TI{FRT.Tub.GAL80.O}101F* suppression of GAL4: dCORL.GAL4 was crossed to *TI{FRT.Tub.GAL80.O}101F*. One-day-old female adult progeny were stained for GFP and dILP2 or dILP5.

(3) Testing *TI{TI}FRT101F* and *TI{FRT.Tub.GAL80.O}101F* recombination: To generate MARCM clones males homozygous for *TI{TI}FRT101F* were mated to females homozygous for *P{ry[+t7.2]=70FLP}3F* and *TI{FRT.Tub.GAL80.O}101F*. Eggs were collected for 10 h and then aged 4 h (egg age at heat shock was 4–14 h). Heat-shock was performed once at 37°C. One-day-old adult female progeny from heat-shocked and not heat-shocked controls were stained for GFP and dILP5 expression.

## Results

### Mitotic recombination resource generation

**Fig. 1.**
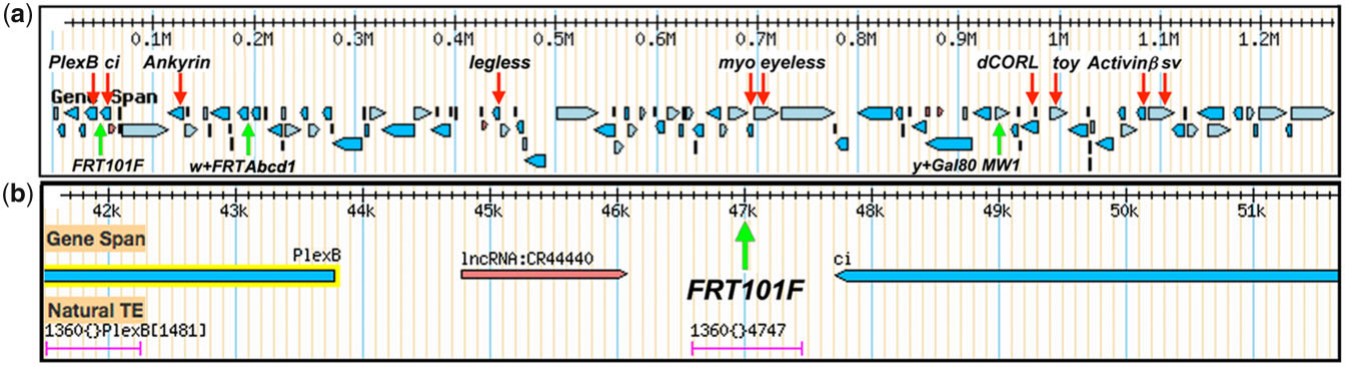
FRT101F location and the genomic organization of the 4th long arm. a) Downloaded from Flybase, the gridline reflects base pair numbering with the centromere to the left. The arm contains 79 protein-coding genes (blue rectangles roughly to scale with transcription direction indicated) and 26 non-coding RNAs (red rectangles drawn similarly). Several well-known genes are identified with red arrows. The insertion sites of 3 transgenes are indicated with green arrows. These are *TI{TI}FRT101F* for mitotic clones as well as *PBac{w[+mC]-WH}Abcd1^f00836^* and *P{y[+t7.7]=ET.GAL80}MW1* for meiotic recombination. Employing GenBank NC_004353.4 (2017) numbering *TI{TI}FRT101F* and *TI{FRT.Tub.GAL80.O}101F* are located at 46,995 bp, *PBac{w[+mC]-WH}Abcd1^f00836^* at 192,043 bp and *P{y[+t7.7]=ET.GAL80}MW1* at 960,138 bp. b) Flybase Gbrowse image of the 101F/102A boundary near the base of the 4th. The location of *TI{TI}FRT101F* and *TI{FRT.Tub.GAL80.O}101F* is shown with a green arrow in the intergenic region between *PlexinB* (transcribed antiparallel from 43,778 bp) and *ci* (transcribed antiparallel to 47,710 bp). The locations of the lncRNA CR44440 (transcribed in parallel to 46,074 bp) and a 1360 transposable element are also indicated.

(1) *TI{TI}FRT101F-DsRed+* and *TI{FRT.Tub.GAL80.O}101F-DsRed+* construction. The genomic organization of the long arm of the 4th is shown in Fig. 1a. The location of well-known genes such as *PlexinB* and *eyeless* are shown as well as 3 transgenes employed to enable mitotic and meiotic recombination on the 4th. A close-up view of the proximal region at the boundary of 101F and 102A between *PlexinB* and *ci* is shown in Fig. 1b. Both *TI{TI}FRT101F-DsRed+* and *TI{FRT.Tub.GAL80.O}101F-DsRed+* were targeted to this region at 46,995 bp. This location was chosen after several attempts at homology directed repair proximal to *PlexinB* at 20,400 bp failed, perhaps due to pericentric heterochromatin.

The predicted lncRNA CR4440 is also present in the *PlexinB* to *ci* intergenic region. *TI{TI}FRT101F-DsRed+* and *TI{FRT.Tub.GAL80.O}101F-DsRed* were targeted between the lncRNA and *ci*, where they would not disrupt any genes. However, unavoidably they are located within a 1360 transposable element capable of nucleating heterochromatin (Sun *et al.* 2004). The 1360 element may explain the failure of *phiC31* integrase to insert attB plasmids into the attP site of transformants for the FRT-attP intermediate. The 1360 element does not impact homology directed repair, α-Tubulin (84B*)* promoter expression of GAL80 or FLP-FRT recombination.

The transgenes for *TI{TI}FRT101F-DsRed+* and *TI{FRT.Tub.GAL80.O}101F-DsRed* both pre- and post-insertion are shown with the intermediate plasmid FRT-attP in Supplementary Figs. 1–4. Transformation efficiency for each of the 3 injected plasmids was between 2% and 6% of surviving G0 flies with the larger *TI{FRT.Tub.GAL80.O}101F-DsRed* at the lower end. After establishing homozygous stocks, DsRed was removed from both transgenes via FLP-FRT intrachromosomal recombination leaving behind a single FRT site.

**Fig. 2.**
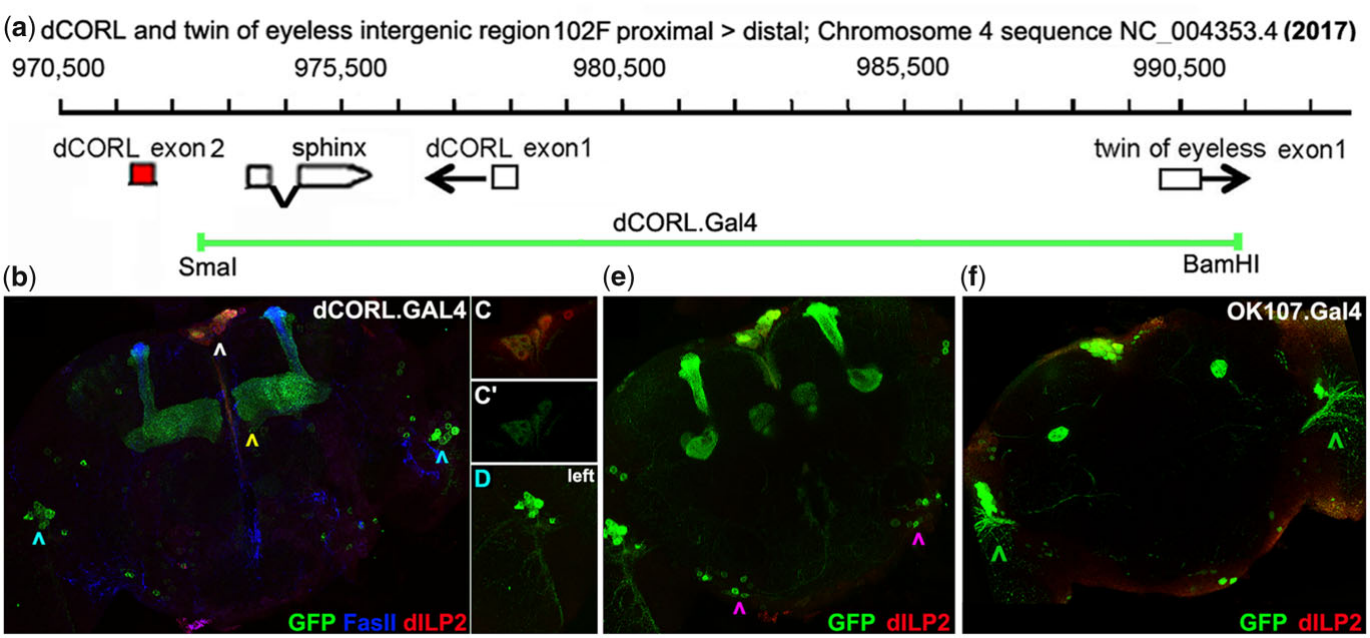
dCORL.GAL4 genomic map and adult brain expression. a) Gridline numbering from Fig. 1. Portions of the intron-exon structure of *dCORL*, *sphinx* and *toy* are shown. White boxes indicate untranslated exons and the red box is translated. Transcription orientation is indicated by an arrow. The green line indicates the region contained in dCORL.GAL4. b–f). One-day-old adult female brains expressing GFP (green), FasII (blue in b only), and dILP2 (red). B) Full stack at 40x (roughly 95 2 µm slices) displaying dCORL.GAL4 driven GFP in 3 regions. dILP2 expressing IPC neurons (white arrowhead), mushroom body α /α’ and β /β’ lobes (yellow arrowhead) and in each lobula plate (blue arrowheads). c, c’) Single slice in 2 colors or GFP alone indicating that most dILP2 IPC neurons express dCORL.GAL4. d) Close-up view of lobula plate cells reveals dendrites projecting ventrally and laterally into the optic lobe. The number, location, and projection trajectory suggest that these cells are visual projection neurons of the vertical system (VS and VS-like neurons). e) Small stack displaying additional sets of 6–7 cells near the posterior edge of the brain. These cells have axons projecting medially and anteriorly (purple arrowheads). f) OK107.GAL4 enhancer trap in *eyeless* is expressed in the same regions as dCORL.GAL4—IPC neurons, mushroom body lobes and lobula plate neurons. However, the number, location and projection trajectory of the OK107.GAL4 lobula plate cells (green arrowheads) suggest that they are not the same as those expressing dCORL.GAL4.

(2) Characterizing dCORL.GAL4 expression. A recent analysis indicated that *dCORL* (*fussel* in Flybase and SKOR2 in mammals; Stinchfield *et al.* 2019) is expressed in every dILP2 producing cell (IPC) in the adult brain and that *dCORL* functions in the activation of dILP2 expression (Tran *et al.* 2018b). A companion study of *dCORL* regulation employing a set of overlapping reporter genes showed that the intergenic region upstream of *dCORL* contains a complex pattern of intertwined stage- and tissue-specific enhancers and repressors for *dCORL* but no enhancers for the adjacent divergently transcribed gene *twin of eyeless* (*toy*; Tran *et al.* 2018a). To further our studies of *dCORL*, we cloned the region between *dCORL* exon2 and *toy* exon2 in front of GAL4 in both directions (Fig. 2a, details Supplementary Figs. 5–7).

The fidelity of adult brain expression from the dCORL.GAL4 chromosome was tested with dILP2 as a marker. dCORL.GAL4 driven GFP expression was co-localized with dILP2 in IPCs (Fig. 2, b and c). However, in each brain 2 or 3 dILP2 expressing cells did not express dCORL.GAL4. We interpret the distinction from universal dILP2 co-expression (as seen before with the dCORL reporter AH.lacZ; Tran *et al.* 2018a) as an effect of the intertwined enhancers and repressors identified previously. This interpretation is supported by lack of complete co-expression of dCORL.GAL4 and AH.lacZ even though AH.lacZ is fully contained within dCORL.GAL4 (Supplementary Figs. 5, 6 b and c).

There are 2 brain regions that express dCORL.GAL4 that do not express dILP2 (Fig. 2, b, d, and e). One is the mushroom body. This neuropil has an enigmatic relationship with *dCORL*. Initial genetic analysis in mushroom body neurons indicated a requirement for *dCORL* downstream of the Baboon receptor for the expression of Ecdysone ReceptorB-1 (Takaesu *et al.* 2012). Subsequently, RNA in situ did not detect *dCORL* in the mushroom body (Tran *et al.* 2018a). As noted above, reporter studies of the region contained in dCORL.GAL4 revealed a complex set of intertwined enhancers and repressors. Taken together, we explain dCORL.GAL4 expression in the mushroom body as consistent with the genetic evidence. Except for the brief period when it is required, *dCORL* is silenced by a repressor not contained in dCORL.GAL4.

To support this hypothesis, we sought to exclude the possibility that dCORL.GAL4 mushroom body expression was due to *sphinx*. This gene is a long noncoding RNA in the *dCORL* first intron (included in dCORL.GAL4; Fig. 2a) that functions in gustatory neurons connected to the antennal lobe of the brain (Chen *et al.* 2011). A *sphinx* RNA in situ was conducted. This was not as simple as it sounds for 2 reasons. First, *sphinx* contains 2 exons, a unique 4th chromosome exon1 and a retro-transposed portion of *ATPsynF* from the 2nd chromosome as exon2. Second, *dCORL* transcript RD originates in the *sphinx* intron and its exon3 occupies the opposite strand of a portion of *sphinx* exon1. These 2 artifacts impact all *sphinx* cDNAs and may have led to the conclusion that *dCORL* functions in gustatory neurons (Rass *et al.* 2019). To avoid artifacts due to *ATPsynF* and *dCORL*, a *sphinx* probe containing only exon1 sequences that are not complementary to *dCORL* transcript RD was generated by PCR (Supplementary Fig. 8a). While a low level of unspliced *dCORL*-RD was identified with the sense strand (compare Supplementary Fig. S8, b and c), no *sphinx* expression was detected with the anti-sense strand in third instar larval brains (Supplementary Fig. 8d).

The second region expressing dCORL.GAL4 but not dILP2 is the bilaterally symmetrical lobula plate (Fig. 2, b, d, and e). Here dCORL.GAL4 is expressed in a small group of cells. Based on their number, position, and projection pattern these dCORL.GAL4 expressing neurons could be a subset of visual projection neurons. The projection pattern of dCORL.GAL4 cells laterally into the optic lobe and posteriorly toward the “bottom” of the optic lobe resembles that of neurons in the vertical coordinate system (VS and VS-like neurons; Scott *et al.* 2002; Boergens *et al.* 2018).

*eyeless* was shown to be expressed in adult IPC neurons where it completely overlaps with dCORL and dILP2 (Tran *et al.* 2018b). That study employed OK107.GAL4 (an enhancer piracy insertion in *eyeless;*Connolly *et al.* 1996). OK107.GAL4 expression was previously noted in IPC neurons, mushroom body and lobula plate (Aso *et al.* 2009; Okamoto *et al.* 2012). The number, position and projection pattern of OK107.GAL4 lobula plate neurons suggest that they are distinct from dCORL.GAL4 lobula plate neurons (Fig. 2f).

**Fig. 3.**
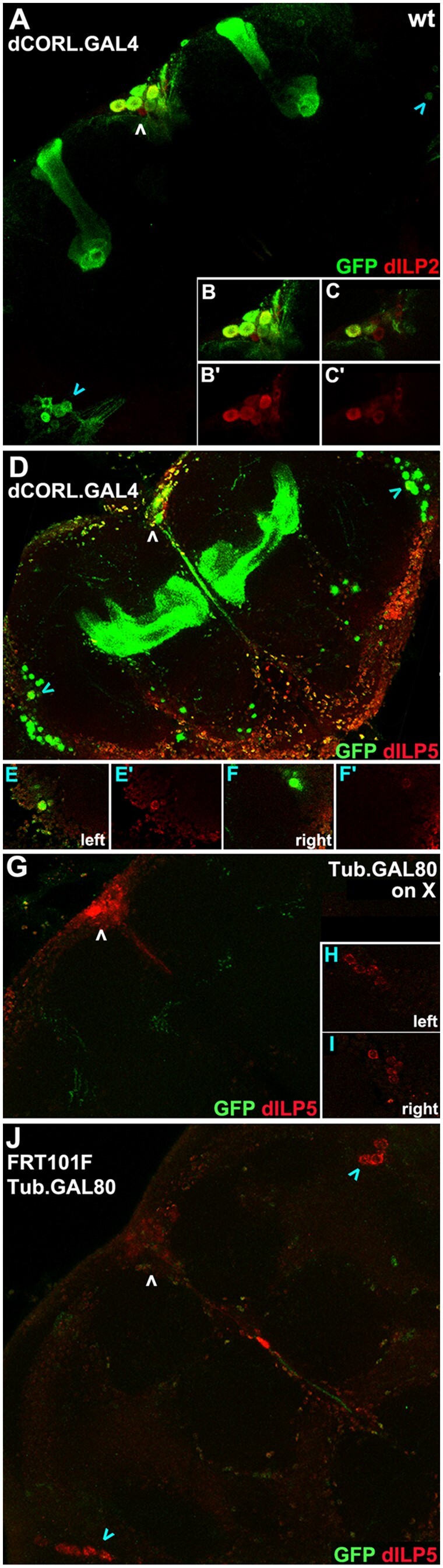
Effective *TI{FRT*.Tub.*GAL80.O}101F* suppression of dCORL.GAL4. One-day-old adult female brains expressing dCORL.GAL4 displaying GFP (green) and dILP2 or dILP5 (red). a) Wild type. Small stack showing GFP and dILP2 co-expression in many but not all IPC neurons (white arrowhead). GFP alone is seen in VS neurons (blue arrowheads) and mushroom body lobes. b, b’, c, c’) IPC neurons in close-up as a small stack or single slice in 2 colors or dILP2 alone documenting co-expression. d) Wild type. Small stack showing GFP and dILP5 co-expression in IPC neurons (white arrowhead) and a pair of VS neurons (blue arrowheads). e, e’, f, f’) Each co-expressing VS neuron in close-up as a single slice in 2 colors or dILP5 alone documenting co-expression. g–i) *P{w[+mC]=Tub.GAL80}LL1.* Tub.GAL80 on X is a positive control for GAL4 suppression. Small stack showing complete suppression of dCORL.GAL4 in IPC (white arrowhead), mushroom body and VS neurons. Low-level GFP expression in 1 or 2 mushroom body axons is present. j) *TI{FRT.Tub.GAL80.O}101F.* Small stack showing complete suppression of dCORL.GAL4 in IPC (white arrowhead), mushroom body and VS neurons (blue arrowheads). Low-level nonspecific GFP expression is present.

(3) Testing *TI{FRT.Tub.GAL80.O}101F* suppression of GAL4. To validate the *TI{FRT.Tub.GAL80.O}101F* chromosome for MARCM we examined its ability to suppress dCORL.GAL4. We conducted side-by-side comparisons of *TI{FRT.Tub.GAL80.O}101F* and its parental line *P{w[+mC]=Tub.GAL80}LL1* (the same Tub.GAL80 plasmid is deployed in both). We included dILP2 and dILP5 as markers in these experiments because dILP5 is expressed in IPC neurons and in cells of the lobula plate (Brent and Rajan 2020). A comparison of dILP2 and dILP5 expression in IPC neurons with dCORL.GAL4 expression confirmed the presence of all 3 (Fig. 3, a–d). Comparison of dCORL.GAL4 and dILP5 in lobula plate cells revealed that a subset of these cells express both (Fig. 3, e and f). We confirmed the distinction between lobula plate OK107.GAL4 neurons and dCORL.GAL4 neurons, as OK107.GAL4 does not overlap with any dILP5 cells in this region.

Suppression of dCORL.GAL4 expression by both *TI{FRT.Tub.GAL80.O}101F* and *P{w[+mC]=Tub.GAL80}LL1* was complete. Only background levels of GFP were visible (Fig. 3, g–j). This result indicates that *TI{FRT.Tub.GAL80.O}101F* could be suitable for generating MARCM clones. We then tested the ability of the FRT sites in *TI{FRT.Tub.GAL80.O}101F* to recombine with *TI{TI}FRT101F* by creating MARCM clones.

(4) Testing *TI{TI}FRT101F* and *TI{FRT.Tub.GAL80.O}101F* recombination. To generate wild-type MARCM clones we assembled a genotype with the *TI{TI}FRT101F* and *TI{FRT.Tub.GAL80.O}101F* homologous 4th chromosomes and the recombinant 2nd chromosome with dCORL.GAL4/UAS.GFP. We applied a single 1-h heat-shock to 4–14 h-old embryos with this genotype and examined 1-day adult female brains. The timing of heat shock was dictated by evidence that each hemisphere of adult IPC neurons originate from a single embryonic neuroblast (Wang *et al.* 2007). This cell is visible at stage 12 (7:20–9:20 h after fertilization) and the IPC neuron complement appears complete by stage 14 (10:20–11:20 h). IPC neuroblast emergence is just after the appearance of 4 mushroom body neuroblasts per hemisphere at stage 9 (3:40–4:20 h) with γ lobe neurons generated during stage 12 (7:20–9:20 h). The other mushroom body lobes are generated postembryonically (Kunz *et al.* 2012). VS neurons derive from a single neuroblast in each hemisphere whose embryonic origin is known only as “before stage 17” (Sen *et al.* 2014). With its neural-specific GFP expression dCORL.GAL4 could reveal heat-shock stimulated, FRT dependent mitotic recombination in either neuroblasts or ganglion mother cells (Scott *et al.* 2002).

**Fig. 4.**
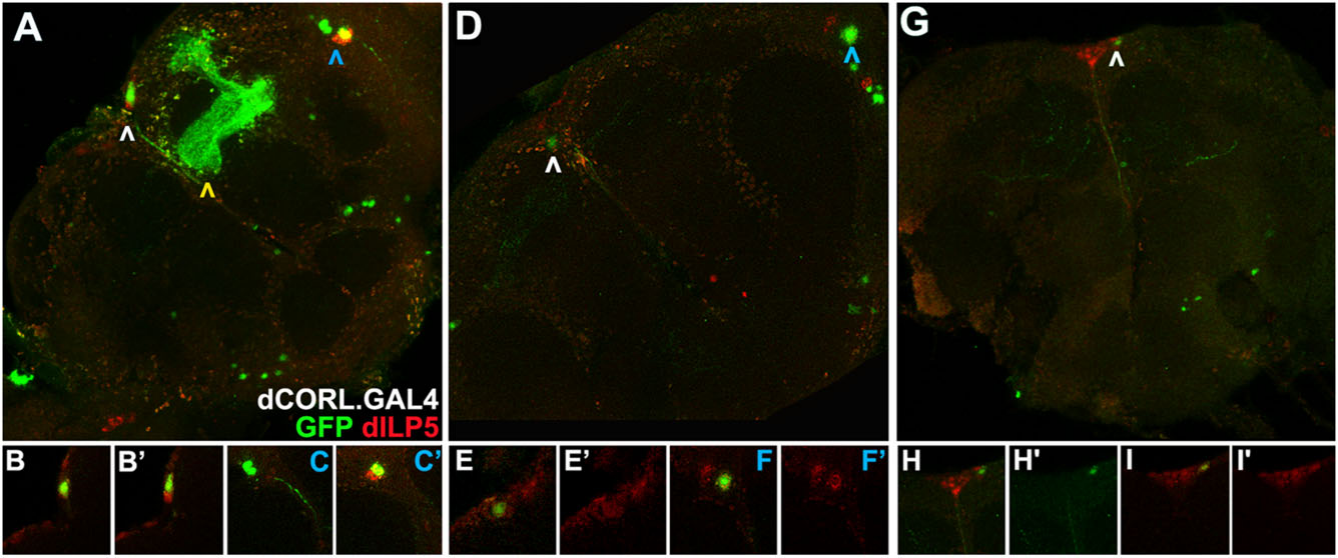
FRT101F enabled dCORL.GAL4 MARCM clones. One-day-old female adult brains with wild type clones resulting from *TI{TI}FRT101F* and *TI{FRT.Tub.GAL80.O}101F* recombination in the presence of dCORL.GAL4. Clones were revealed by GFP expression (green) in comparison to dILP5 (red). Top row is 2 color brain full stacks. Bottom row is single slices (or a small stack in h, h'). a) Brain with multi-cell neuroblast clones in 3 regions. Yellow arrowhead indicates a large mushroom body clone encompassing α/α’ and β/β’ neurons. White arrowhead indicates an IPC clone of 2 neurons. Blue arrowhead indicates a clone of 3 VS neurons. b, b') Slices demonstrating that each GFP expressing IPC neuron co-expresses dILP5. c, c') Slices documenting that one of the three GFP expressing VS neurons co-expresses dILP5. The pair that does not express dILP5 is in a slice dorsal to the slice with the co-expressing neuron. d) Brain with single ganglion mother cell clones in 2 regions. White arrowhead indicates a single IPC neuron clone. Blue arrowhead indicates a single VS neuron clone. e, e') Slice of the IPC clone in 2 colors and red only revealing co-expression of dILP5. f, f') Slice of the VS clone in 2 colors and red only demonstrating co-expression of dILP5. g) Brain with a single ganglion mother cell clone in an IPC neuron. h, h') Small stack of the IPC clone in 2 colors and green only documenting its location. i, i') Slice of the IPC clone in 2 colors and red only demonstrating co-expression of dILP5.

We identified multiple adult brains with MARCM clones. At one end of the range, there were brains with multi-cell clones in 3 regions suggesting heat shock induced mitotic recombination occurred independently in several embryonic neuroblasts (Fig. 4, a–c). In the middle of the range, there were brains with a single cell clone in 2 or 3 regions suggesting recombination occurred independently in several embryonic ganglion mother cells (Fig. 4, d–f). At the other end of the range, there were brains with a single cell clone in 1 region suggesting recombination occurred in just 1 ganglion mother cell (Fig. 4, g–i). In MARMC clone brains, GFP was seen in each region associated with dCORL.GAL4: mushroom body, IPC, and VS cells.

Having multiple brains with clones allowed us to calculate the efficiency of FRT101F enabled mitotic recombination. MARCM clone relative frequency corresponded roughly to the number of embryonic neuroblasts and the number of subsequent cell divisions. The 4 neuroblasts that together generate the 2,500 neurons of the adult mushroom body (Technau 1984) produced clones that were consistently larger than the other regions and were visible in nearly 40% of brains. The single IPC neuroblast that generates roughly 8–9 neurons produced 1–3 cell clones in approximately 9% of brains. The single VS neuroblast that generates roughly 9–10 neurons produced only single-cell clones, at the same rate as IPC clones. With this demonstration of mitotic recombination and a reasonable frequency of wild-type MARCM clones, the ability to conduct MARCM studies of mutations in genes on the 4th is not far off.

### Meiotic recombination resource generation

To facilitate the ability to place mutations on the *TI{TI}FRT101F* chromosome, a step necessary for conducting MARCM studies on the 4th, we enabled meiotic recombination in the female germline. As a result, the process from pre-existing mutation to MARCM clones on the 4th is now the same as for the other fly chromosomes. In addition, our crossing schemes are easily adopted by investigators for application to their favorite gene/mutation.

**Fig. 5.**
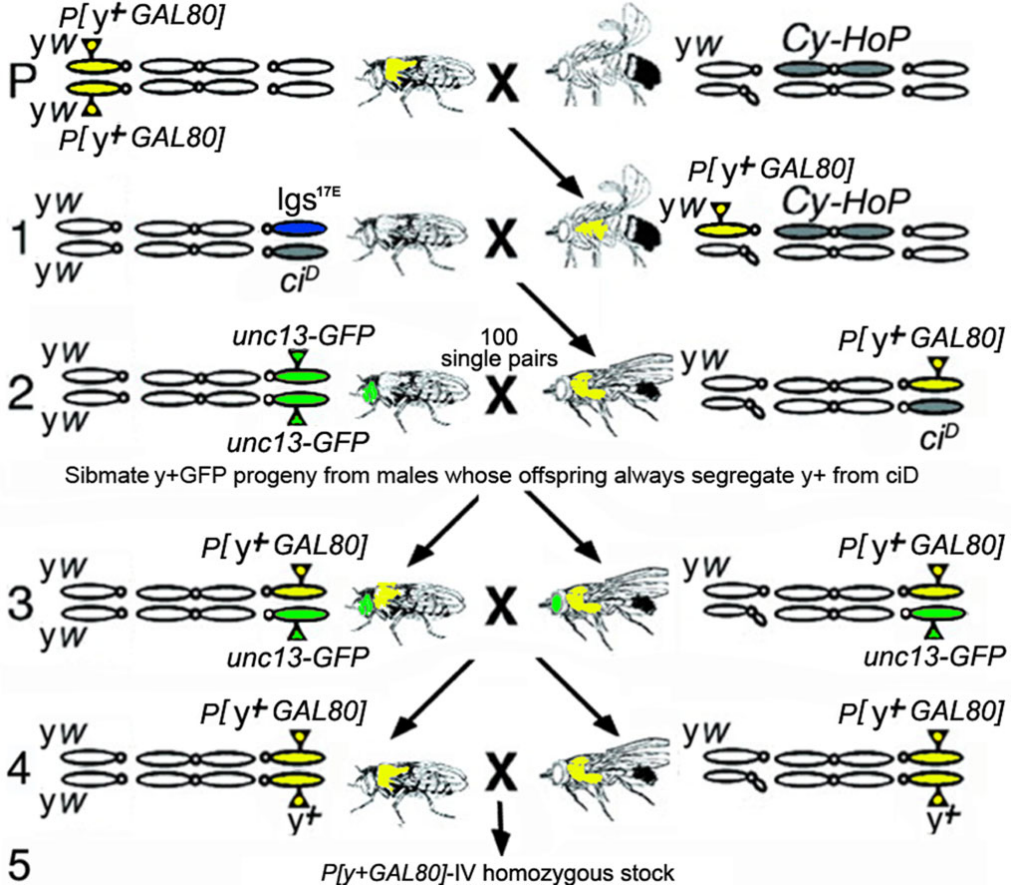
Jumping a *y+* transgene off the X onto the 4th. Crossing scheme with females on the left displaying their X, 2nd and 4th chromosomes. Parental: Females homozygous for *P{y[+t7.7]=ET.GAL80* (shown as yellow body color) on the X were mated to males with an immobile source of P element transposase (HoP) inserted on the *CyO* 2nd chromosome balancer. Line 1: Males with *P{y[+t7.7]=ET.GAL80* and transposase could have a potential jump in their germline. Line 2: Scoring male to male transmission of *y*+ eliminated the parental X and any local jumps. Line2 crosses with 248 single *y+* males to females with the visible marker unc13: GFP (shown as green eyes) led to the identification of 2 insertions of *P{y[+t7.7]=ET.GAL80* on the 4th in line 3. These 2 were identified by consistent segregation of *y+* away from *ci^D^* in the progeny of line 2. Line 3: Progeny heterozygous for *y+* and unc13: GFP from the males with progeny displaying perfect segregation away from *ci^D^* were sibmated. Line 4: Further sibmating created a homozygous stock for genomic DNA extraction, inverse PCR and sequencing to confirm the presence of *P{y[+t7.7]=ET.GAL80* on the 4th.

**Fig. 6.**
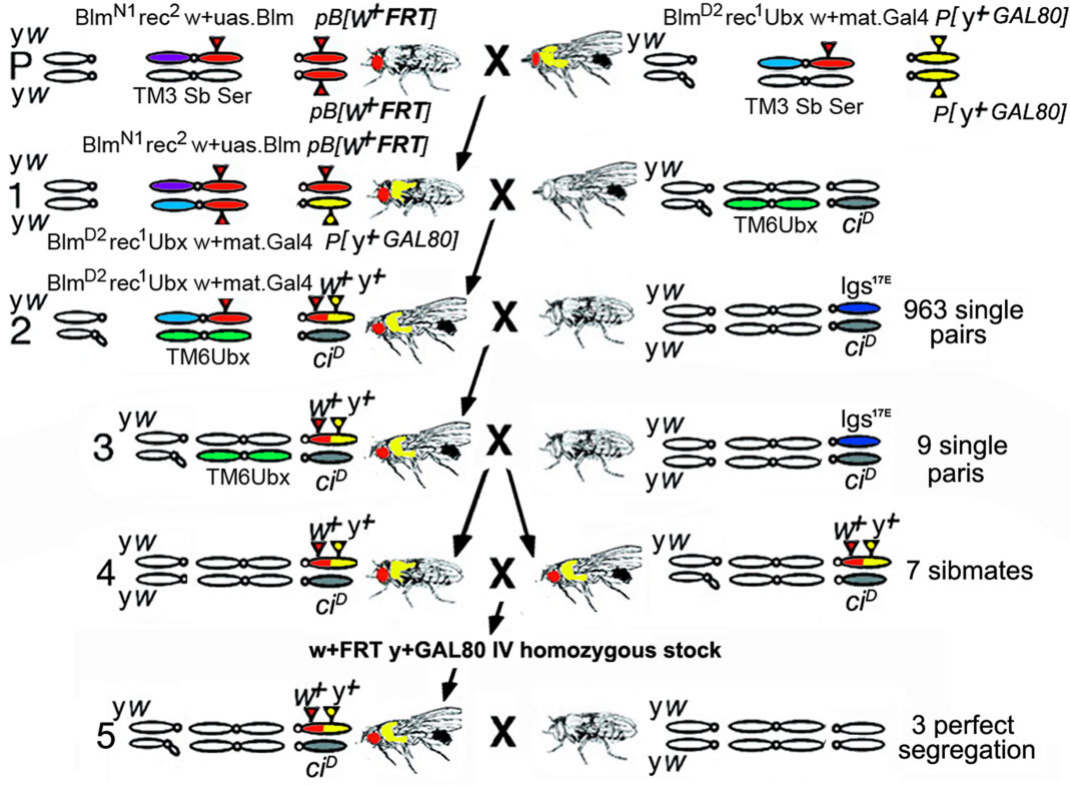
*Blm rec* enabled 4th chromosome meiotic recombination and recovery of 3 recombinant chromosomes. Crossing scheme with males showing a dark abdomen displaying their X, 3rd and 4th chromosomes. Colors: *w+* (red), *y+* (yellow), *Blm^N1^* (purple), *Blm^D2^* (light blue), *TM6-Ubx* (green), and *ci^D^* (gray). Parental: *Blm^N1^ rec^2^ P{w[+mW]=UASp.Blm}* heterozygous and *PBac{w[+mC]-WH}Abcd1^f00836^* homozygous females (left) were crossed to *Blm^D2^ ry rec^1^ Ubx^bx34e^ P{w[+mW]mat-α.GAL4}* heterozygous and *P{y[+t7.7]=ET.GAL80}MW1* homozygous males (right). From the progeny, recombination competent females were collected with the genotype *Blm rec* double mutant plus *PBac{w[+mC]-WH}Abcd1^f00836^* and *P{y[+t7.7]=ET.GAL80}MW1* transheterozygous. Line 1: Recombination competent females (left) were crossed to males carrying *TM6-Ubx* and *ci^D^*. Line 2: Candidate recombinant males (left) were collected with *PBac{w[+mC]-WH}Abcd1^f00836^* and *P{y[+t7.7]=ET.GAL80}MW1* over *ci^D^* also displaying an enhanced *TM6-Ubx* phenotype due to the presence of *Ubx^bx34e^* to eliminate the *Blm^N1^ rec^2^ P{w[+mW]* *=* *UASp.Blm}* chromosome. Nine hundred and sixty-three candidate males were individually crossed to *ci^D^* heterozygous females. Line 3: 9 candidate recombinant males (left) with *PBac{w[+mC]-WH}Abcd1^f00836^* and *P{y[+t7.7]=ET.GAL80}MW1* over *ci^D^* and a normal *TM6-Ubx* phenotype were collected after eliminating the *Blm^D2^ ry rec^1^ Ubx^bx34e^ P{w[+mW]mat-α.GAL4}* chromosome. The 9 candidate recombinant males were individually crossed to heterozygous *ci^D^* females. Line 4: From the progeny of 7 surviving males, *PBac{w[+mC]-WH}Abcd1^f00836^* and *P{y[+t7.7]=ET.GAL80}MW1* over *ci^D^* males and females were collected and sibmated for a heterozygous stock. Line 4 progeny: 7 sets of *PBac{w[+mC]-WH}Abcd1^f00836^* and *P{y[+t7.7]=ET.GAL80}MW1* progeny that did not display *ci^D^* were sibmated for 7 homozygous recombinant stocks. Line 5: *PBac{w[+mC]-WH}Abcd1^f00836^* and *P{y[+t7.7]=ET.GAL80}MW1* and *ci^D^* males (left) from each of the 7 stocks in line 4 were crossed to *yw* females to test for 100% co-segregation of *PBac{w[+mC]-WH}Abcd1^f00836^* and *P{y[+t7.7]=ET.GAL80}MW1* from *ci^D^*. Progeny from 3 recombinant stocks (#332, #343, and #360) displayed perfect segregation away from *ci^D^*.

(1) X to 4th transposition: To demonstrate the frequency of transposition from the X to the 4th we jumped a *yellow+* marked transgene *P{y[+t7.7]=ET.GAL80}* (Potter *et al.* 2010). Prior to starting the scheme, all stocks were placed into an *yw* background (Fig. 5). Screening for male-to-male transmission of *y+* eliminated the original chromosome and any local jumps. Screening 248 *y+* males yielded 2 homozygous viable *y+* insertions (0.8%) that faithfully segregated from the 4th chromosome marker *ci^D^*. These insertions were named *P{y[+t7.7]=ET.GAL80}MW1* and *P{y[+t7.7]=ET.GAL80}MW2*. Inverse PCR showed that *MW1* is located at 960,138 bp in the non-coding RNA CR44030 that has no known function (Fig. 1a). *MW2* is located at 660,477 bp in the untranslated first exon of *gawky*, a micro-RNA associated protein required for nuclear divisions during embryonic development.

(2) *Blm rec* enabled meiotic recombination: To demonstrate the frequency of 4th recombination we planned to recombine the *y+ P{y[+t7.7]=ET.GAL80}MW1* transgene onto a *white+* FRT 4th chromosome (Fig. 6). For the experiment, we chose the homozygous viable 4th chromosome insertion *PBac{w[+mC]-WH}Abcd1^f00836^* (Fig. 1a). This is a piggyBac transgene (Bellen *et al.* 2011) bearing a single FRT inserted at 192,043 bp in the first intron of the *ABC-type fatty-acyl-CoA transporter1*.

It is well known that the 4th has no recombination. One current hypothesis is that the absence stems from double-strand break repair. During meiosis these breaks are repaired only via the Class I pathway as non-crossovers (Hatkevich *et al.* 2017). However, a *Bloom syndrome helicase* (*Blm*) and *recombination defective* (*rec*) double mutant genotype allows meiotic recombination on the 4th in females. Two pre-existing mutations on a single 4th chromosome (*ci^D^* at 47,710 bp and *sv^Spa^* at 1,088,798 bp; distance 1,041,088 bp, Fig. 1a) were separated at a frequency of roughly 0.3% (10 out of 3106; Hatkevich *et al.* 2017). We employed this genotype with the inverse goal, to recombine *PBac{w[+mC]-WH}Abcd1^f00836^* and *P{y[+t7.7]=ET.GAL80}MW1* (distance 768,095 bp, Fig. 1a) onto a single chromosome. Importantly, our goal went beyond simply counting recombinants; we wanted to capture the recombinant chromosome for use in clonal analysis. Thus, we needed to avoid false positives not considered previously such as a triplo-4 genotypes (Stenberg *et al.* 2009) or illegitimate recombination between the *P{y[+t7.7]=ET.GAL80}MW1* bearing 4th and any *w+* chromosomes in the crossing scheme. Prior to starting the scheme, all stocks were put into a *yw* background.

First, we created the 2 stocks that when crossed would yield the *Blm rec* double mutant recombination competent females (Fig. 6, parental). One stock was heterozygous for the recessive alleles *Blm^N1^* and *rec^2^* on the 3rd with *PBac{w[+mC]-WH}Abcd1^f00836^* homozygous on the 4th. The other stock contained 3 recessive alleles *Blm^D2^*, *rec^1^*, and *Ubx^bx34e^* on the 3rd with *P{y[+t7.7]=ET.GAL80}MW1* homozygous on the 4th. In addition, to compensate for the very low fertility of the double mutant females, a *w+ Blm* related transgene was additionally present on each 3rd: [*w+*; UASp.Blm] with *Blm^N1^* and [*w+*; mat-α.GAL4-VP16] with *Blm^D2^*. These transgenes insure the presence of maternal Blm during oogenesis, such that the oocytes containing meiotic recombination products survive to be fertilized and become recombinant adults (Hatkevich *et al.* 2017). Mating of these lines created females with a transheterozygous *Blm rec* double mutant genotype that were also heterozygous for *PBac{w[+mC]-WH}Abcd1^f00836^* and *P{y[+t7.7]=ET.GAL80}MW1* on the 4th (Fig. 6, line 1).

Second, the key to the recombination scheme was having a suitable tester chromosome. In our case, 4 tester chromosomes were needed due to the presence of *w+* transgenes on both *Blm* mutant 3rd chromosomes. In the first 2 generations of the recombination competent female’s progeny, these *w+* transgenes would obscure the presence of *PBac{w[+mC]-WH}Abcd1^f00836^* when linked to *P{y[+t7.7]=ET.GAL80}MW1* on a recombinant 4th. Both *w+ Blm* 3rd chromosomes needed to be eliminated before the third test (4th chromosome co-segregation of *PBac{w[+mC]-WH}Abcd1^f00836^* and *P{y[+t7.7]=ET.GAL80}MW1*) could be conducted. In a fourth test, we would eliminate potential false positives such as triplo-4 genotypes and illegitimate recombination.

We scored for the presence of *w+* and *y+* in male progeny of recombination competent females. We conducted the first tester cross (Fig. 6, line 1) in mass and the second tester cross (Fig. 6, line 2) in batches of 100 single pairs per week with a total of 963 *w+ y+* males. The first test cross eliminated the *Blm^N1^ rec^2^ P{w[+mW]=UASp.Blm}* chromosome. The second test cross eliminated the *Blm^D2^ ry rec^1^ Ubx^bx34e^ P{w[+mW]mat-α.GAL4}* chromosome. Among the F2 progeny 9 candidate *w+ y+* recombinant males were collected (0.94% of 963 initial males). This frequency surprised us since the distance between *PBac{w[+mC]-WH}Abcd1^f00836^* and *P{y[+t7.7]=ET.GAL80}MW1* is 30% shorter than the distance between *ci^D^* and *sv^Spa^* (768,095bp vs 1,041,088bp; Fig. 1a). Possible explanations are that *ci^D^* is an inversion locally suppressing recombination in the prior study and the inclusion of false positives in our study.

The third test cross was conducted in single pairs (Fig. 6, line 3). It eliminated both the *Ubx* balancer employed in the first test cross and allowed for formal evaluation of co-segregation of *PBac{w[+mC]-WH}Abcd1^f00836^* and *P{y[+t7.7]=ET.GAL80}MW1*. Of our 9 recombinants, 2 died and the progeny of the 7 surviving males were sibmated (Fig. 6, line 4) for a potential homozygous *PBac{w[+mC]-WH}Abcd1^f00836^* and *P{y[+t7.7]=ET.GAL80}MW1* stock. Males from each stock were then retested (Fig. 6, line 5; fourth test) for formal co-segregation of *PBac{w[+mC]-WH}Abcd1^f00836^* and *P{y[+t7.7]=ET.GAL80}MW1* away from *ci^D^*. Here, 3 recombinant 4th chromosomes showed perfect co-segregation of *w+* and *y+* away from *ci^D^* (stocks from males #332, #343, and #360) Overall, from a set of 963 single males we recovered 9 initial recombinant candidates (0.94%), yielding 3 demonstrated recombinant candidates (0.31%) containing both *PBac{w[+mC]-WH}Abcd1^f00836^* and *P{y[+t7.7]=ET.GAL80}MW1*.

(3) *y* wing clones: To validate the recombinant *PBac{w[+mC]-WH}Abcd1^f00836^* and *P{y[+t7.7]=ET.GAL80}MW1* chromosome we showed that the FRT will recombine when homozygous. The FRT experiment was conducted in flies with a *yw* background that were heterozygous for one of the 3 recombinant chromosomes and their parent *PBac{w[+mC]-WH}Abcd1^f00836^* chromosome. Heat-shock induced FLP recombinase was able to generate *y* clones (indicating loss of *P{y[+t7.7]=ET.GAL80}MW1* in those cells), formally demonstrating the presence of *P{y[+t7.7]=ET.GAL80}MW1* on the *PBac{w[+mC]-WH}Abcd1^f00836^* chromosome. The 3 recombinant 4th chromosomes each yielded easily visible *y* clones on their wings, legs, and notum. Negative controls were the 4 failed recombinant candidates. These gave no *y* clones in any part of the body in over 200 flies examined for each.

**Fig 7.**
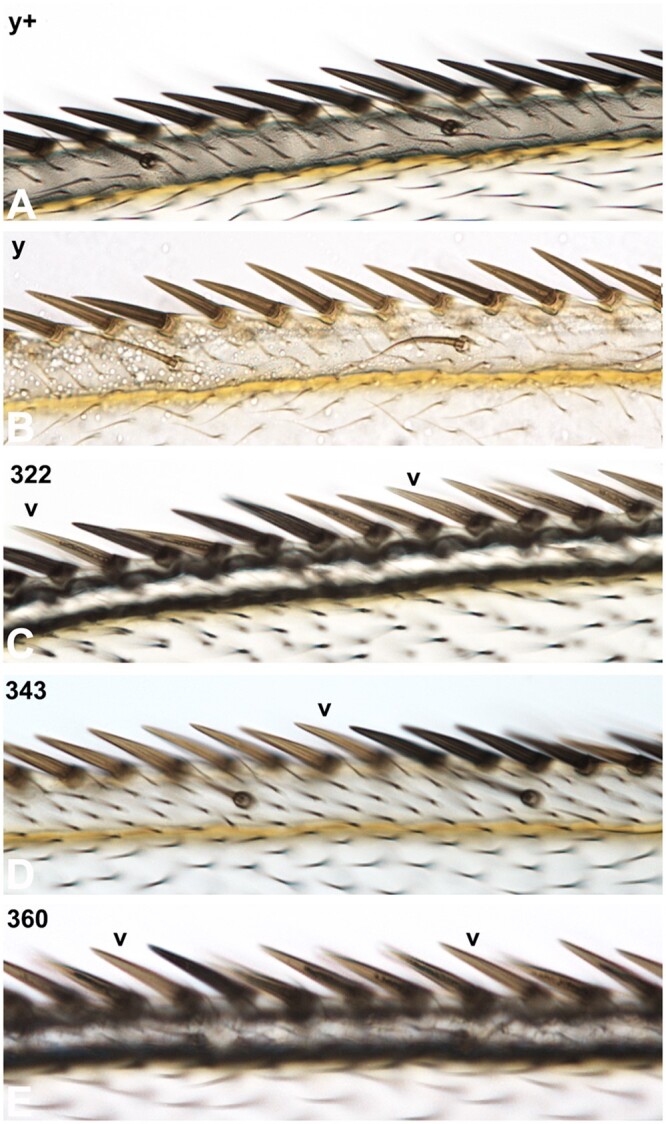
*y* wing margin bristle clones validate the 3 recombinant 4th chromosome. The anterior-dorsal region of a female wing is shown with proximal to the left. Focus is on the dense row of stout mechanosensory bristles along the dorsal-ventral margin and the wide-spaced row of thin chemosensory bristles on the dorsal surface above longitudinal vein one. a) Wild type wing with normal dark brown bristles. b) Genotypically *y* wing showing light brown bristles. c) *Blm rec* recombinant #322 wing shows 2 small clones of a single *y* mechanosensory bristle (arrowheads). d) Recombinant #343 wing shows a large clone with 8 y mechanosensory bristles (arrowhead indicates the most distal bristle in the clone with 13 additional *y* bristles out of view to the left; 21 y bristles total). e) Recombinant #360 wing is similar to #322 with 2 small clones of a single *y* mechanosensory bristle (arrowheads).

We illustrate the experiment with *y* clones in wing margin stout mechanosensory bristles in comparison to wild type and *y* mutants (Fig. 7, a and b). As above for MARCM clones in the brain, there was a range of clone phenotypes. Recombinants #322 and #360 showed multiple single bristle clones suggesting recombination in a sensory organ precursor cell (Fig. 7, c and e). Recombinant #343 showed a large clone of over 20 bristles indicating recombination in a neuroblast (Fig. 7d). The occurrence of meiotic recombination on the 4th is confirmed.

## Discussion

Here, we describe multiple new strains enabling mitotic and meiotic recombination on the 4th chromosome. These resources will facilitate the genetic analyses of poorly understood genes on this chromosome. These analyses are necessary to expand our understanding of metazoan development and homeostasis. The strains will shortly be publicly available, are amenable to studies by undergraduates and can be employed to generate unmarked and MARCM clones for several types of 4th mutations.

### Mitotic recombination resource generation

For those who wish to place a protein-coding mutation on the *TI{TI}FRT101F* chromosome, a comprehensive collection of vetted guide RNA stocks is available. Stocks with guide RNAs for every 4th chromosome protein-coding gene plus one with maternal Cas-9 can be ordered (Kondo and Ueda 2013; National Institute of Genetics, Mishima Japan). In our experience, the crossing method with guide RNA stocks is more efficient and cheaper than commercial injection of guide RNA plasmids.

Briefly, in the crossing method females homozygous for Cas9 and *TI{TI}FRT101F-DsRed+* are crossed to males homozygous for a specific guide RNA. In triple heterozygote progeny females double, strand breaks are generated in the target gene by Cas9. Then imperfect DNA repair leads to small deletions that create frameshifts 66% of the time. These females are mated to males heterozygous for *ci^D^*. Progeny males containing candidate mutant chromosomes are recovered by tracking DsRed and *ci^D^*. Candidate mutant males are mated individually to females heterozygous for *ci^D^*. Male and female progeny from each candidate male are sibmated to determine homozygous viability. If feasible a homozygous mutant stock (also homozygous for *TI{TI}FRT101F-DsRed+*) is created. PCR of each mutant with vetted primers (also available from the National Institute of Genetics, Mishima) is followed by removal of DsRed from *TI{TI}FRT101F-DsRed+* by standard methods. In our hands, the crossing method yielded 4 unique mutations in our favorite 4th gene among the sequenced progeny of 20 candidate males.

For those who prefer “off the shelf” stocks for their studies, the 4th Chromosome Resource Project is currently generating chromosomes with *TI{TI}FRT101F* and a mutation in each protein-coding gene on the 4th for the community. These lines will be described elsewhere.

### Meiotic recombination resource generation

To generate a recombinant 4th chromosome we exploited the *Blm rec* recombination inducing genotype. This is the first application of this genotype to link two 4th chromosome markers together and recover the recombinant chromosome for future use. Our crossing scheme can be recreated in any lab using the *Blm^N1^ rec^2^ PBac{w[+mC]-WH}Abcd1^f00836^* stock and the companion *Blm^D2^ rec^1^* stock carrying a *y+* transgene in a gene of interest on the 4th. The scheme allows generation of a recombinant 4th chromosome with a functional *PBac{w[+mC]-WH}Abcd1^f00836^* and a *y+* marked transgene without employing CRISPR. The recombinant chromosome can then be employed to make *y* clones when *in trans* to the parental *PBac{w[+mC]-WH}Abcd1^f00836^* chromosome.

One example is to create *y* clones in the adult antenna with a homozygous lethal *y+* MiMIC in PMCA, a plasma membrane bound calcium ATPase. Numerous *y+* Minos and MiMIC insertions on the 4th were produced by the Gene Disruption Project (Bellen *et al.* 2011; Venken *et al.* 2011). *y+* MiMIC inserted in a coding intron allows application of conversion technology to an eGFP protein tag or a T2A.GAL4 protein truncation mutant allele (Nagarkar-Jaiswal *et al.* 2015a, 2015b). If the goal is to make clones, then conversion must be applied to a *y+* MiMIC after recombination onto the FRT chromosome as the *y+* marker is lost during conversion. Then the eGFP protein or T2A.GALl4 allele can be analyzed in unmarked clones.

There are 93 MiMIC insertions on the 4th with hits in 51 protein-coding genes. Among these are insertions that create a mutant allele directly (e.g. *PlexA^MB09499^*) and others in a coding intron capable of being converted (e.g. *dCORL^MI03207^*). As above, for those who prefer “off the shelf” stocks for their studies, the 4th Chromosome Resource Project is currently converting all intronic MiMICs to eGFP and T2A.GAL4 for the community. To date, 34 converted lines have been donated to the Bloomington Drosophila Stock Center with more conversions in progress.

Combining the 2 sets of resources described here, an investigation can conduct a meiotic recombination experiment with a *w+* or *y+* marked transgene and *TI{TI}FRT101F-DsRed+*. Following the creation of a recombinant 4th chromosome, DsRed would be removed. Then mitotic recombination dependent MARCM clones could be generated with the recombinant chromosome in trans to *TI{FRT.Tub.GAL80.O}101F* with a GAL4 line and heat-shock FLP.

In summary, to overcome well-known obstacles to studying genes on the 4th chromosome we created new strains. For mitotic recombination, we generated a chromosome with an FRT very near the centromere in 101F and a derivative that carries FRT101F with a distal ubiquitously expressed GAL80 transgene. This pair of chromosomes enables both unmarked and MARCM clones. For meiotic recombination, we demonstrate that a *Blm* and *recombination defective* double mutant genotype can create recombinant 4th chromosomes via female meiosis. All strains will be available to the community via the Bloomington Drosophila Stock Center. Additional resources for studies of the 4th chromosome are in preparation and will also be made available. The goal of the 4th Chromosome Resource Project is to accelerate the genetic analysis of protein-coding genes on the 4th, including the 44 genes with no demonstrated function. Studies of these previously inaccessible but largely conserved genes will close longstanding gaps in our knowledge of metazoan development and physiology.

## Data availability

The authors affirm that all data necessary for confirming the conclusions of the article are present within the article, figures, and Supplemental Information. Parental stocks will soon be available from Bloomington Drosophila Stock Center. Prior to deposition detailed schemes and stocks are available to qualified investigators upon request from the corresponding author.

Supplemental material is available at *G3* online.

## Supplementary Material

jkac019_Supplementary_Data
