# Supplementary material for: New resources for the Drosophila 4th chromosome: FRT101F enabled mitotic clones and Bloom syndrome helicase enabled meiotic recombination
Source: G3 (Bethesda). 2022 Jan 27;12(4):jkac019. doi: 10.1093/g3journal/jkac019 (PMC8982423; doi:10.1093/g3journal/jkac019)
Supplement: jkac019_Supplementary_Data [file jkac019_supplementary_data.pdf]

Supplemental Information

Molecular Biology Methods

I.A. Construction of *TI{TI}FRT101F-DsRed+* and *TI{FRT.Tub.GAL80.O}101F-DsRed+*

1) attP-FRT: Two rounds of Gibson assembly were utilized to make arm1-attP-FRT-DsRed-FRT-arm2. Prior to the first assembly, the homology arm1 region of the 4th was PCR amplified from genomic DNA (Table S1, rows 1/2). After *EcoRI* and *NotI* digestion, both the arm1 PCR fragment and pHD-DsRed-attP-w+ (Addgene #80898) were mixed with Gibson oligonucleotides (Table S2, rows1/2) and a synthetic fragment containing the distal region of arm1 proximal attP and FRT sequences ending in a *NotI site* (Table S3, row 1). Prior to the second assembly, the homology arm2 region of the 4th was PCR amplified from genomic DNA (Table S1, rows 3/4). After *BglII* and *XhoI* digestion of the arm2 PCR fragment and the modified pHD-DsRed-attP-w+ constructed above, they were mixed with two Gibson oligonucleotides (Table S2, rows 3/4 where line 4 starts with an *XhoI* overhang TCGA) and a synthetic fragment beginning in a *BglII* site that contains an FRT plus the proximal region of arm2 (Table S3, row 2). Post-assembly the sequences of the proximal attP, the two FRT sites and DsRed were verified (Table S4, rows 1-5) prior to injection. In transformants, visible DsRed and loss of *w+* indicated successful homology directed repair and the transgene was named *TI{TI}FRT101F-DsRed+*.

2) FRT-attP: Two rounds of standard cloning were necessary to make arm1-FRT-DsRed-FRT-attP-arm2. In the first round, the arm1 region of the 4th was PCR amplified from genomic DNA (Table S1, rows 1/2). After *EcoRI* and *NotI* digestion, the fragment was ligated into the *EcoRI* and *NotI* sites of pHD-DsRed-attP-w+. In the second round, an FRT with a distal attP attached to the proximal region of arm2 was ligated into a *BglII* and *PstI* digest of the pHD-DsRed-attP-w+ arm1 construct above. This was accomplished by sequential insertion of two fragments into the pHD-DsRed-attP-w+ arm1 construct. The first inserted fragment was a *BglII* and *HindIII* digest of a PCR fragment (Table S1, rows 3/4) generated from a synthetic segment of FRT and attP sequences cloned into pUC57 (Table S3, row 3). The second inserted fragment was a *HindIII* and *PstI* digest of the homology arm2 region of the 4th amplified from genomic DNA (Table S1,

rows 5/6). Post-assembly the sequences of the two FRT sites, the attP site and DsRed were verified (Table S4, rows 1-5) prior to injection. This intermediate was employed to build FRT-Tub.GAL80 as described below.

3) FRT-Tub.GAL80: One round of Gibson assembly was followed by site directed mutagenesis and standard cloning to make arm1-FRT-DsRed-FRT-Tub.GAL80-arm2. Prior to the assembly, a fragment containing the ubiquitous α-Tubulin (84A) promoter linked to GAL80-polyA was generated by PCR from pCaSpeR4 (Addgene #17748; Table S2, rows 5-8). In the assembly, an in-house generated plasmid containing attP and an MCS was digested with *XbaI* and *NotI,* then mixed with the Tub.GAL80 PCR product and Gibson oligonucleotides (Table S2, rows 5-8). This assembled plasmid was then subjected to site-directed mutagenesis (QuikChange II XL kit, Agilent Technologies) to introduce unique *AvrII* and *SphI* sites flanking Tub.GAL80-polyA (Table S5, rows 1-4). The FRT-attP plasmid above containing arm1-FRT-DsRed-FRT-attP-arm2 was also mutagenized to introduce unique *AvrII* and *SphI* sites around the attP sequence (Table S5, rows 5-8). Post-mutagenesis, the two FRT sites in the FRT-attP plasmid were checked by sequence (Table S4, rows 1/3 and 4/5). Both mutagenized plasmids were digested with *AvrII* and *SphI*. The attP fragment in the FRT-attP plasmid was replaced by a fragment containing Tub.GAL80 from the other plasmid. Both FRT sites, DsRed, the α-Tubulin promoter, GAL80 and both arm junctions were verified by sequence (Table S4, rows 1-9). In transformants, visible DsRed and loss of *w+* indicated successful homology directed repair and the transgene was named *TI{FRT.Tub.GAL80.O}101F-DsRed+*.

I.B. Oligonucleotides for *TI{TI}FRT101F-DsRed+* & *TI{FRT.Tub.GAL80.O}101F-DsRed+*

Lowercase nucleotides contain restriction sites that do not anneal to the template or for Gibson oligonucleotides contain regions that will anneal to the adjacent fragment of the assembly.

Table S1. 4th chromosome and synthetic DNA PCR primers

EcoRI arm1 for	5′ gtcgtcgaattcGCACGAGCTCTTGTGACATTG 3′
arm1 FRT Not rev	5′ gtcgtcgcggccgcGAAGTTCCTATACTTTC TAGAGAATAGGAACTTCCGTGTTGTCCCGTGGGTAC 3′
BglII FRT attP for	5′ cagagatctGAAGTTCCTATTCTCTAGAAAGTATAG 3′
HindIII FRT attP rev	5′ atcaagcttAACCCCTTGTGTCATGTCGG 3′
HindIII arm2 for	5′ gtcaaagcttGGGAGGCCATTGCAATTG 3′
PstI arm2 rev	5′ cagctgcagGTTTGTGACACCCAAGCG 3′

Table S2. Gibson assembly oligonucleotides

Gib arm1 for	5′ cgctgaagcaggtggGCACGAGCTCTTGTGACATTG 3′
Gib arm1 rev	5′ gtgggtaccgattattacaTATATTACATATAATTAA TTATTAACATAAATATAAATATGTAAACG 3′
Gib arm2 for	5′ gtgatactatgtacatagATTCGGCAACTACTACTTTCAAG
Gib arm2 rev	5′ tcgattgacggaagagccGTTTGTGACACCCAAGCG 3′
Gib Tub_fwd	5′gcgtaagcttcgtacgtagcGAATTCGATATCAAGCTTGCAC3′
Gib Tub_rev	5′ tgtagtccatTTCACGCTGTGGATGAGG 3′
Gib GAL80_fwd	5′ acagcgtgaaATGGACTACAACAAGAGATCTTC 3′
Gib GAL80_rev	5′ tcgtcgacactagtggatctAGGCCTTCTAGTGGATCC 3′

Table S3. Synthetic DNA fragments (GeneScript)

arm1 - attP - FRT - *NotI* Legend: part of arm 1 attP FRT part of vector (*NotI*)

TGTAATAATCGGTACCCACGGGACAACACGGTAGTGCCCCAACTGGGGTAACCTTTGAGTTCTC
TCAGTTGGGGGCGTAGGAAGTTCCTATTCTCTAGAAAGTATAGGAACTTCCGGCCGCGGACATA
TGCACACCTGCGATCGTAG

***BglII* - FRT - arm2** Legend: (*BglII*) part of vector FRT part of arm 2

CGAAGTTATAGAAGAGCACTAGTAAAGATCGAAGTTCCTATTCTCTAGAAAGTATAGGAACTTC
GGGAGGCCATTGCAATTGTAATTCACATGTCGCAAGCAGAATACTCTAATGGCCTAT

FRT - attP in pUC57 Legend: part of vector FRT attP part of arm2

CGAAGTTATAGAAGAGCACTAGTAAAGATCGAAGTTCCTATTCTCTAGAAAGTATAGGAACTTC
CCCAGGTCAGAAGCGGTTTTCGGGAGTAGTGCCCCAACTGGGGTAACCTTTGAGTTCTCTCAGT
TGGGGGCGTAGGGTCGCCGACATGACACAAGGGGTTGGGAGGCCATTGCAATTGTAATTCACAT
GTCGCAAGCAGAATACTCTAATGGCCTATATTCTAATATAAAGTCATTTTTAAAATTTATTTTG
TGATACTATGTACATAG

Table S4. Transgene construction verification sequencing primers

IV target 11F	5′ CGCCTCTTGAATTAGCTACCG 3′	arm1->DsRed
IV target 15F	5′ GAAACAAGAAATGGATATTCGCC 3′	arm1->DsRed
mCherry[a] rev	5′ TTGGTCACCTTCAGCTTGG 3′	DsRed->arm1
IV target 04R	5′ GGCTCCAGGCTCTAACG 3′	arm2->Sv40
EBV rev[b]	5′ GTGGTTTGTCCAAACTCATC 3′	Sv40->arm2
Tub out rev	5′ GTGGTCTGACTACTTGTAAATGTTAG 3′	Tub->DsRed
GAL80 1F	5′ CATCCACAGCGTGAAGG 3′	GAL80->arm2
GAL80 2F	5′ CTCAAGGCTATATCGGCG 3′	GAL80->arm2
GAL80 3F	5′ GCCAATGGACAACAAGCTC 3′	GAL80->arm2

a. mCherry anneals within DsRed to sequence out of DsRed across an FRT and into arm1.
b. EBV rev anneals within SV40 polyA to sequence out of DsRed across an FRT and into arm2.

Table S5. Site directed mutagenesis oligonucleotides

Tub *AvrII* top	5′ GTACTGTCTGCAGCCTAGGCTTCGTACGTAGCG 3′
Tub *AvrII* bot	5′ CGCTACGTACGAAGCCTAGGCTGCAGACAGTAC 3′
GAL80 *SphI* top	5′ GTATCTTATCATGTCTGCATGCACTAGAAGGCCTAGATC 3′
GAL80 *Sph*I bot	5′ GATCTAGGCCTTCTAGTGCATGCAGACATGATAAGATAC 3′
attP *AvrII* top	5′ GTATAGGAACTTCCCTAGGTCAGAAGCGGTTTTCG 3′
attP *AvrII* bot	5′ CGAAAACCGCTTCTGACCTAGGGAAGTTCCTATAC 3′
attP *SphI* top	5′ GCCGACATGACACAAGGCATGCAGCTTGGGAGGCCATTG 3′
attP *SphI* bot	5′ CAATGGCCTCCCAAGCTGCATGCCTTGTGTCATGTCGGC 3′

Table S6. CRISPR Cas9 guide RNA oligonucleotides

Sense oligo guide 1	5′ CTTCGCCTCCCCGTGTTGTCCCGT 3′
Antisense oligo guide 1	5′ AAACACGGGACAACACGGGGAGGC 3′
Sense oligo guide 2	5′ CTTCGGCCTCCCCGTGTTGTCCCG 3′
Antisense oligo guide 2	5′ AAACCGGGACAACACGGGGAGGCC 3′

Table S7. Additional sequencing primers for inserted *TI{TI}FRT101F-DsRed+*

attP for	5′ GTAACCTTTGAGTTCTCTCAG 3′	arm1->FRT
attP on IV 14R	5′ GTCGCTCCGTAGACGAAGC 3′	FRT->arm1
DsRed rev	5′ CGCTCGTACTGCTCCAC 3′	arm2->FRT

Table S8. Additional sequencing primers for inserted *TI{FRT.Tub.GAL80.O}101F-DsRed+*

GAL80 4F	5′ GAGTCATGCTGTGTTTAGTAATAG 3′	GAL80->arm2
arm2-2 rev	5′ GCGTTGCATACTCGTGGG 3′	GAL80->arm2
5′arm1-2 for	5′ GCTGCCGTGAAGCTAAGAATGC 3′	arm1->genome
attP rev	5′ CTGAGAGAACTCAAAGGTTACC 3′	arm1->genome
3′arm2-3 rev	5′ GACTCATCGATGACTAGCTTGCC 3′	arm2->genome

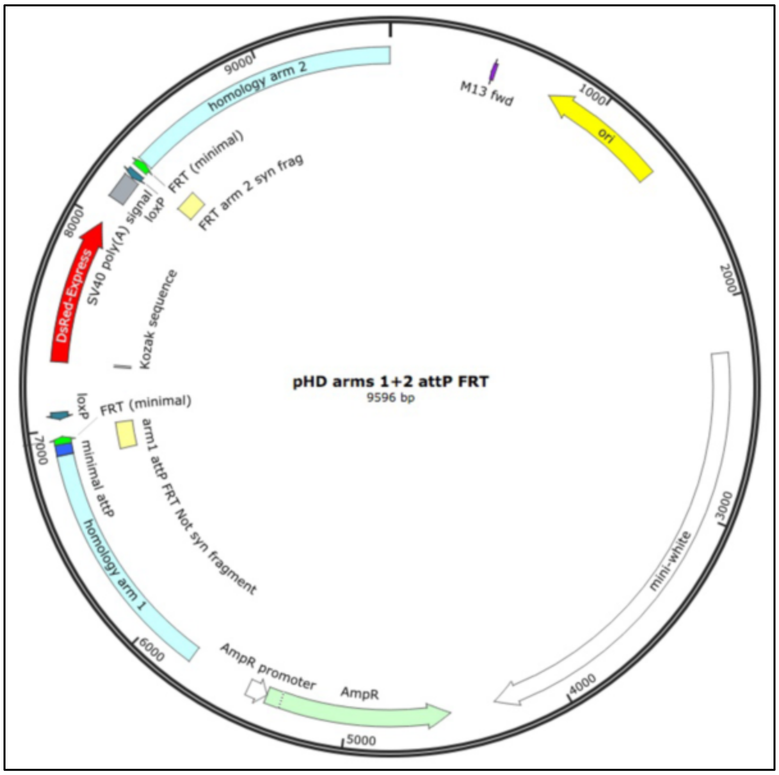

Fig. S1. ***TI{TI}FRT101F-DsRed+* transgene map.** This transgene is also known as attP-FRT. The complete plasmid with the two homology arms (arm1 is proximal; light blue bars), two unidirectional FRT sites (green arrows) separated by a DsRed marker (red arrow) are shown. Additional features such as two synthesized fragments (yellow bars), 2 loxP sites (dark blue arrows), a minimal attP site proximal to one FRT (dark blue bar) and optimization sequences such as Kozak and SV40 polyA are shown. The inserted sequence is shown below. After identification of a successful insertion in 101F, DsRed was removed leaving behind a single functional FRT site as shown in Fig. S4.

```
attP FRT DsRed target site          Homology arm1 CCACGGGACAACACG
GTAGTGCCCCAACTGGGGTAACCTTTGAGTTCTCTCAGTTGGGGGCGTAGGAAGTTCCTATTCT
CTAGAAAGTATAGGAACTTCCGGCCGCGGACATATGCACACCTGCGATCGTAGTGCCCCAACTG
GGGTAACCTTTGAGTTCTCTCAGTTGGGGGCGTAGATAACTTCGTATAATGTATGCTATACGAA
GTTATCGTACGGGATCTAATTCAATTAGAGACTAATTCAATTAGAGCTAATTCAATTAGGATCC
AAGCTTATCGATTTCGAACCCTCGACCGCCGGAGTATAAATAGAGGCGCTTCGTCTACGGAGCG
ACAATTCAATTCAAACAAGCAAAGTGAACACGTCGCTAAGCGAAAGCTAAGCAAATAAACAAGC
GCAGCTGAACAAGCTAAACAATCGGCTCGAAGCCGGTCGCCACCATGGCCTCCTCCGAGGACGT
CATCAAGGAGTTCATGCGCTTCAAGGTGCGCATGGAGGGCTCCGTGAACGGCCACGAGTTCGAG
ATCGAGGGCGAGGGCGAGGGCCGCCCCTACGAGGGCACCCAGACCGCCAAGCTGAAGGTGACCA
AGGGCGGCCCCCTGCCCTTCGCCTGGGACATCCTGTCCCCCCAGTTCCAGTACGGCTCCAAGGT
GTACGTGAAGCACCCCGCCGACATCCCCGACTACAAGAAGCTGTCCTTCCCCGAGGGCTTCAAG
TGGGAGCGCGTGATGAACTTCGAGGACGGCGGCGTGGTGACCGTGACCCAGGACTCCTCCCTCC
AGGACGGCTCCTTCATCTACAAGGTGAAGTTCATCGGCGTGAACTTCCCCTCCGACGGCCCCGT
AATGCAGAAGAAGACTATGGGCTGGGAGGCGTCCACCGAGCGCCTGTACCCCCGCGACGGCGTG
CTGAAGGGCGAGATCCACAAGGCCCTGAAGCTGAAGGACGGCGGCCACTACCTGGTGGAGTTCA
AGTCCATCTACATGGCCAAGAAGCCCGTGCAGCTGCCCGGCTACTACTACGTGGACTCCAAGCT
GGACATCACCTCCCACAACGAGGACTACACCATCGTGGAGCAGTACGAGCGCGCCGAGGGCCGC
CACCACCTGTTCCTGTAGGGGCCGCGACTCTAGATCATAATCAGCCATACCACATTTGTAGAGG
TTTTACTTGCTTTAAAAAACCTCCCACACCTCCCCCTGAACCTGAAACATAAAATGAATGCAAT
TGTTGTTGTTAACTTGTTTATTGCAGCTTATAATGGTTACAAATAAAGCAATAGCATCACAAAT
TTCACAAATAAAGCATTTTTTTCACTGCATTCTAGTTGTGGTTTGTCCAAACTCATCAATGTAT
CTTAACCGGTATAACTTCGTATAATGTATGCTATACGAAGTTATAGAAGAGCACTAGTAAAGAT
CGAAGTTCCTATTCTCTAGAAAGTATAGGAACTTCGGGAGGC Homology arm2
```

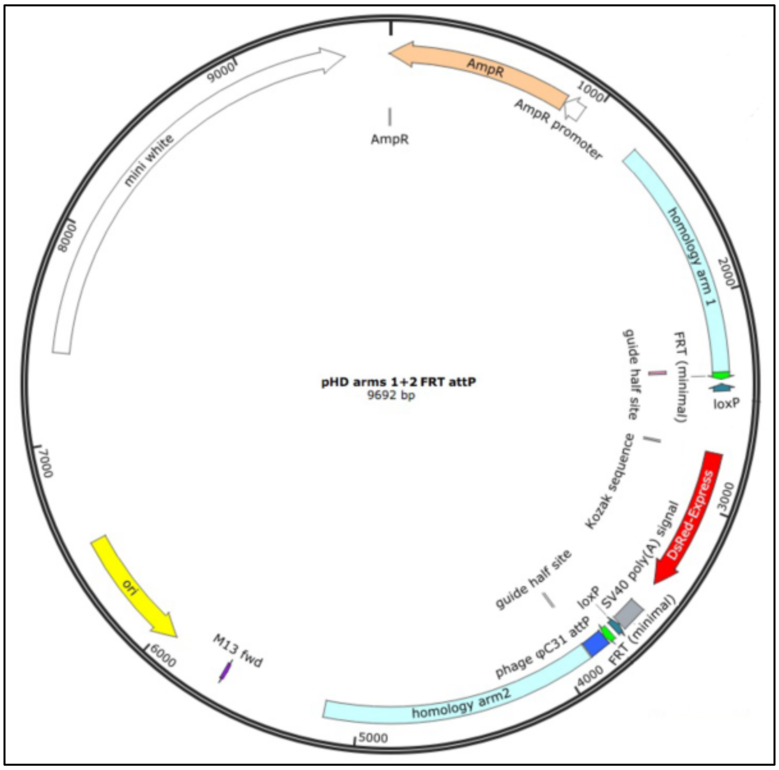

Fig. S2. Intermediate FRT-attP transgene map. This transgene is also known as FRT-attP. The complete plasmid with the two homology arms (arm1 is proximal; light blue bars), two unidirectional FRT sites (green arrows) separated by a DsRed marker (red arrow) are shown. Additional features such as two loxP sites (dark blue arrows), a minimal attP site distal to one FRT (dark blue bar) and optimization sequences such as Kozak and SV40 polyA are shown. The inserted sequence is shown below. The attP was non-functional and this transgene was employed to create *TI{FRT.Tub.GAL80.O}101F-DsRed+* from *TI{TI}FRT101F-DsRed+*.

DsRed FRT attP target site **Homology arm1 CCACGGACAACACG**
GAAGTTCCTATTCTCTAGAAAGTATAGGAACTTCGCGGCCGCGGACATATGCACACCTGCGATC
GTAGTGCCCCAACTGGGGTAACCTTTGAGTTCTCTCAGTTGGGGGCGTAGATAACTTCGTATAA
TGTATGCTATACGAAGTTATCGTACGGGATCTAATTCAATTAGAGACTAATTCAATTAGAGCTA
ATTCAATTAGGATCCAAGCTTATCGATTTCGAACCCTCGACCGCCGGAGTATAAATAGAGGCGC
TTCGTCTACGGAGCGACAATTCAATTCAAACAAGCAAAGTGAACACGTCGCTAAGCGAAAGCTA
AGCAAATAAACAAGCGCAGCTGAACAAGCTAAACAATCGGCTCGAAGCCGGTCGCCACCATGGC
CTCCTCCGAGGACGTCATCAAGGAGTTCATGCGCTTCAAGGTGCGCATGGAGGGCTCCGTGAAC
GGCCACGAGTTCGAGATCGAGGGCGAGGGCGAGGGCCGCCCCTACGAGGGCACCCAGACCGCCA
AGCTGAAGGTGACCAAGGGCGGCCCCCTGCCCTTCGCCTGGGACATCCTGTCCCCCCAGTTCCA
GTACGGCTCCAAGGTGTACGTGAAGCACCCCGCCGACATCCCCGACTACAAGAAGCTGTCCTTC
CCCGAGGGCTTCAAGTGGGAGCGCGTGATGAACTTCGAGGACGGCGGCGTGGTGACCGTGACCC
AGGACTCCTCCCTCCAGGACGGCTCCTTCATCTACAAGGTGAAGTTCATCGGCGTGAACTTCCC
CTCCGACGGCCCCGTAATGCAGAAGAAGACTATGGGCTGGGAGGCGTCCACCGAGCGCCTGTAC
CCCCGCGACGGCGTGCTGAAGGGCGAGATCCACAAGGCCCTGAAGCTGAAGGACGGCGGCCACT
ACCTGGTGGAGTTCAAGTCCATCTACATGGCCAAGAAGCCCGTGCAGCTGCCCGGCTACTACTA
CGTGGACTCCAAGCTGGACATCACCTCCCACAACGAGGACTACACCATCGTGGAGCAGTACGAG
CGCGCCGAGGGCCGCCACCACCTGTTCCTGTAGGGGCCGCGACTCTAGATCATAATCAGCCATA
CCACATTTGTAGAGGTTTTACTTGCTTTAAAAAACCTCCCACACCTCCCCCTGAACCTGAAACA
TAAAATGAATGCAATTGTTGTTGTTAACTTGTTTATTGCAGCTTATAATGGTTACAAATAAAGC
AATAGCATCACAAATTTCACAAATAAAGCATTTTTTTCACTGCATTCTAGTTGTGGTTTGTCCA
AACTCATCAATGTATCTTAACCGGTATAACTTCGTATAATGTATGCTATACGAAGTTATAGAAG
AGCACTAGTAAAGATCT**GAAGTTCCTATTCTCTAGAAAGTATAGGAACTTCCCCAGGTCAGAAG**
CGGTTTTCGGGAGTAGTGCCCCAACTGGGGTAACCTTTGAGTTCTCTCAGTTGGGGGCGTAGGG
TCGCCGACATGACACAAGGGGTTAAGCTT**GGGAGGC Homology arm2**

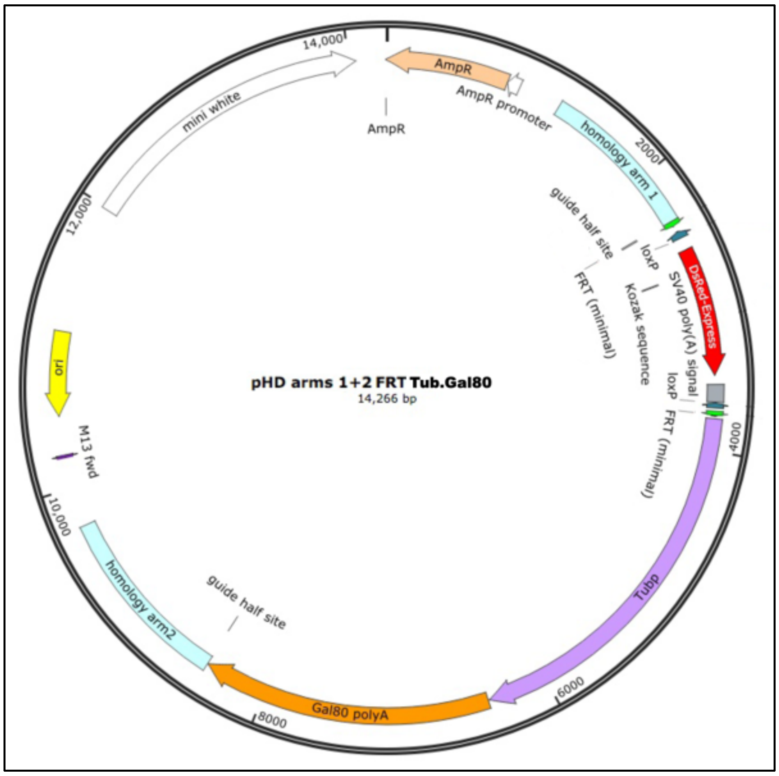

Fig. S3. ***TI{FRT.Tub.GAL80.O}101F-DsRed+*** **transgene map.** This transgene is also known as FRT-Tub.GAL80. The complete plasmid with the two homology arms (arm1 is proximal; light blue bars), two unidirectional FRT sites (green arrows) separated by a DsRed marker (red arrow) are shown. Additional features such as the α-Tubulin (84B) promoter (purple arrow), the GAL80 coding sequence (orange arrow), 2 loxP sites (dark blue arrows) and optimization sequences such as Kozak and SV40 polyA are shown. The inserted sequence is shown below. After identification of a successful insertion in 101F, DsRed was removed leaving behind a single functional FRT site as shown in Fig. S4.

DsRed FRT Tub GAL80 target site Homology arm1 **CCACGGGACAACACG**
GAAGTTCCTATTCTCTAGAAAGTATAGGAACTTCGCGGCCGCGGACATATGCACACCTGCGATC
GTAGTGCCCCAACTGGGGTAACCTTTGAGTTCTCTCAGTTGGGGGCGTAGATAACTTCGTATAA
TGTATGCTATACGAAGTTATCGTACGGGATCTAATTCAATTAGAGACTAATTCAATTAGAGCTA
ATTCAATTAGGATCCAAGCTTATCGATTTCGAACCCTCGACCGCCGGAGTATAAATAGAGGCGC
TTCGTCTACGGAGCGACAATTCAATTCAAACAAGCAAAGTGAACACGTCGCTAAGCGAAAGCTA
AGCAAATAAACAAGCGCAGCTGAACAAGCTAAACAATCGGCTCGAAGCCGGTCGCCACCATGGC
CTCCTCCGAGGACGTCATCAAGGAGTTCATGCGCTTCAAGGTGCGCATGGAGGGCTCCGTGAAC
GGCCACGAGTTCGAGATCGAGGGCGAGGGCGAGGGCCGCCCCTACGAGGGCACCCAGACCGCCA
AGCTGAAGGTGACCAAGGGCGGCCCCCTGCCCTTCGCCTGGGACATCCTGTCCCCCCAGTTCCA

GTACGGCTCCAAGGTGTACGTGAAGCACCCCGCCGACATCCCCGACTACAAGAAGCTGTCCTTC
CCCGAGGGCTTCAAGTGGGAGCGCGTGATGAACTTCGAGGACGGCGGCGTGGTGACCGTGACCC
AGGACTCCTCCCTCCAGGACGGCTCCTTCATCTACAAGGTGAAGTTCATCGGCGTGAACTTCCC
CTCCGACGGCCCCGTAATGCAGAAGAAGACTATGGGCTGGGAGGCGTCCACCGAGCGCCTGTAC
CCCCGCGACGGCGTGCTGAAGGGCGAGATCCACAAGGCCCTGAAGCTGAAGGACGGCGGCCACT
ACCTGGTGGAGTTCAAGTCCATCTACATGGCCAAGAAGCCCGTGCAGCTGCCCGGCTACTACTA
CGTGGACTCCAAGCTGGACATCACCTCCCACAACGAGGACTACACCATCGTGGAGCAGTACGAG
CGCGCCGAGGGCCGCCACCACCTGTTCCTGTAGGGGCCGCGACTCTAGATCATAATCAGCCATA
CCACATTTGTAGAGGTTTTACTTGCTTTAAAAAACCTCCCACACCTCCCCCTGAACCTGAAACA
TAAAATGAATGCAATTGTTGTTGTTAACTTGTTTATTGCAGCTTATAATGGTTACAAATAAAGC
AATAGCATCACAAATTTCACAAATAAAGCATTTTTTTCACTGCATTCTAGTTGTGGTTTGTCCA
AACTCATCAATGTATCTTAACCGGTATAACTTCGTATAATGTATGCTATACGAAGTTATAGAAG
AGCACTAGTAAAGATCTGAAGTTCCTATTCTCTAGAAAGTATAGGAACTTCCCTAGGCTTCGTA
CGTAGC**GAATTCGATATCAAGCTTGCACAGGTCCTGTTCGATAACGTCGTACTCGGGAATCACC**
TTTAACTTGTCCGCTATTGGGTATTTGTCCTTAAACACATTCGTAATCTCTCCCACAATCATTG
CCCTGGCCTCTGCAATGTAAAAAGTATTTATAAAATATCGCTGGCTCTAAAGGAAACATATTAA
TCAAAGTATTGAGGCTTTATCAATCTAACATTTACAAGTAGTCAGACCACAATTTTTAGACTGC
ACTTCTGCGTAAGTAAATGAAATGAAATGGTGACCTACTCTTGGGTAGCCGTACTGTTATATGC
TGACGAGCCTTCACCTGGGCCTGCTTTTTCTCCTGTTTCGTCTGATGCGTCGGATGCGGCTGGA
CTGGCTGATTGTTGGGATTGGGTTTTGGATGCTGCTCCGAAAGGTGGGCCACAGCTTTTGCGAT
GGTGGTGCCCACTATGCGGTTGCTGCAGGAACTACAGCTGAATGTTCGCTTTATTGTCTGCAGC
TCCTGGGGCTGGACGAAGGCCACGCCCTCACCCGGTGCGGCCATCTCCTGCGGCAAAACGTATG
TGGGATTCCTGTCCAAGTAGCTCTCGATGCCCACCATAATGCGCTCCAGCAGGTTGGCCAGGTG
CGGGTCTAGCTGCGGCGGATTCGTCGTCTGGTTCACTGAGTTTAGGGTCTGCAGAAAGAAGTGT
GCCTCCATGGTAAGCGGATTCATCTGCACGATCAGCGGGCTTTAGTCTAAAAATTCGAATGATA
CAAACTCGTACTCACGTAAAGCAGTTTGTTTGGCATCATCTCGGCCATCGTCTTATTCTTCTTC
GTGGCCTTTGTTACCGGAGCGGCTATGTTGGGCGCGGTGGAGCCCGGAAGCTGAAAGGTTAATC
GAATGCGTTAACTTTTCTGCAACTCGAAAGTTTGCCGCCTTTGTTCGACTGCCAATAACTGTTG
ATTCGAAAATTCGAATCGAAGCGGTTGAATTTCGTAGGGTGGCCAACTACACCAAAGTTCGCCG
GCGGTGTATGCATGTGAGTGTGAGTGTGACAGTGGGTGTACGTGTGCTTTAAGCGACACAATCG
CACCGCGATGTACATATTTTTGTAGTAATGTGTGTGCATTTTTCGTCCGGATCACGGCTCCATA
AAATAAATCATATCACAGGGCTCAGGGATTTGTGAGTAGAAGTTCCCGCCACTTAAACGTCACT
TGTGAAATCGCCATCGACGGTCAACTTTTACTCACACTGGTCTTTCGCGGACGGACCGTCTCAA
AGTACTGCCTTTCTGCGTTGGAAAACATCGCCTTTTTCGTCCAAAAGGAGTCCCCAGGTTCGAT
CCGCATGGCGTTGTGCGTGCGTGCCTTTCTTTTCAAATGATTACGGCTATTAACTTGGGGGCGT
TAAGTTGGAAACACGTAAATTGCAGACTGCGATTAGAGTGACCATGAGTAGGAGTTCAAAATCT
CCTGACATCATTTTCTTAAAACCTGCTTTGTTTTTACATTTCTATTTAATATAACTCCTATTT
GAATAAAAAAACAAAACAAGTTTAGATGTTAAGATATTAACTACATCCTTTGCTCCAAAGGGAG
AGGGGAAGTTATGGAGTTAATTAATTTGCTGTTGGAAATCAATATGGAGTCAGAAATATAATGA
TTTACTAAACCTTATTGAATCGGTAACGATGCGAATTTATATTAAAATAGCTTTTATGAAACAT
TCAACAAAAATATTATTAATGTTGGCCCACTTTAGCAACCGGTTAGGTCTACCGGTTGGGCAAG
CAAAGATTCACGCCCTGGTTCGAGTCCCAACTAGTCCTGCAAAATACCGCAGCAAGTTTTAGAG
AGACCAAGTGCCATTACCTCTCCCACTTCAGTTATCGGTTATGCGGCGTTTAAGTCGACAGCTT
GCCGTCTCTAGCTCCGGTGCCTATATAAAGCAGCCCGCTTTCCACATTTCATATTCGTTTTACG
TTTGTCAAGCCTCATAGCCGGCAGTTCGAACGTATACGCTCTCTGAGTCAGACCTCGAAATCGT
AGCTCTACACAATTCTGTGAATTTTCCTTGTCGCGTGTGAAACACTTCCAATAAAAACTCCTGC
AGGTGAGTACTTTAAAAAAAAATCTAGTGAAATAATGCTGAAAAGAAATTTGTGTGGGCAAAAT
TCAATGGGCAAAAACGCGATGCGGCTTTTTCTCAAAATGGCGGCCGGCCTGCGTTTTTTCCTCA

AAAGTGATGACGTCATGCCTGTTTTTTTTTTTTTGTTCGCAATGAGGAATGGCTCTTAAAATCT
ACTAGATAAAAAAAATATTCATTATTTCTATGCTGCTGGAACGCTTCATTAATCTTAAAAATTC
TAAATTCGGTTACCATGATACTTCGACGCATAACTGTAGATTTTGGATAGAATTAAAGAGAAAA
TGGCGAGAGAGTAAAATTCCGGCGTCGGCAAAGTAGAGCAAAAAAATCAGTATACCATTTAGCT
ACCTCTCTCACTCGCACGCAGTGCCGGCTCAAGTTGGGCGCGGCTCTGCAATTATCGATTTTCT
TGGGGTGTGTAACTAATCATCCGTTTTCCCTTCCTCCTCATCCACAGCGTGAAGGTACCCGCCC
GGGGATCAGATCCGCGGCCGCAACATGGACTACAACAAGAGATCTTCGGTCTCAACCGTGCCTA
ATGCAGCTCCCATAAGAGTCGGATTCGTCGGTCTCAACGCAGCCAAAGGATGGGCAATCAAGAC
ACATTACCCCGCCATACTGCAACTATCGTCACAATTTCAAATCACTGCCTTATACAGTCCAAAA
ATTGAGACTTCTATTGCCACCATTCAGCGTCTAAAATTGAGTAATGCCACTGCTTTTCCCACTT
TAGAGTCATTTGCATCATCTTCCACTATAGATATGATAGTGATAGCTATCCAAGTGGCCAGCCA
TTATGAAGTTGTTATGCCTCTCTTGGAATTCTCCAAAAATAATCCGAACCTCAAGTATCTTTTC
GTAGAATGGGCCCTTGCATGTTCACTAGATCAAGCCGAATCCATTTATAAGGCTGCTGCTGAAC
GTGGGGTTCAAACCATCATCTCTTTACAAGGTCGTAAATCACCATATATTTTGAGAGCAAAAGA
ATTAATATCTCAAGGCTATATCGGCGACATTAATTCGATCGAGATTGCTGGAAATGGCGGTTGG
TACGGCTACGAAAGGCCTGTTAAATCACCAAAATACATCTATGAAATCGGGAACGGTGTAGATC
TGGTAACCACAACATTTGGTCACACAATCGATATTTTACAATACATGACAAGTTCGTACTTTTC
CAGGATAAATGCAATGGTTTTCAATAATATTCCAGAGCAAGAGCTGATAGATGAGCGTGGTAAC
CGATTGGGCCAGCGAGTCCCAAAGACAGTACCGGATCATCTTTTATTCCAAGGCACATTGTTAA
ATGGCAATGTTCCAGTGTCATGCAGTTTCAAAGGTGGCAAACCTACCAAAAAATTTACCAAAAA
TTTGGTCATTGACATTCACGGTACCAAGGGAGATTTGAAACTTGAAGGCGATGCCGGCTTCGCA
GAAATTTCAAATCTGGTCCTTTACTACAGTGGAACTAGAGCAAACGACTTCCCGCTAGCCAATG
GACAACAAGCTCCTTTAGACCCGGGGTATGATGCAGGTAAAGAAATCATGGAAGTATATCATTT
ACGAAATTATAATGCCATTGTGGGTAATATTCATCGACTGTATCAATCTATCTCTGACTTCCAC
TTCAATACAAAGAAAATTCCTGAATTACCCTCACAATTTGTAATGCAAGGTTTCGATTTCGAAG
GCTTTCCCACCTTGATGGATGCTCTGATATTACACAGGTTAATCGAGAGCGTTTATAAAAGTAA
CATGATGGGCTCCACATTAAACGTTAGCAATATCTCGCATTATAGTTTATAATCTAGAGGATCT
TTGTGAAGGAACCTTACTTCTGTGGTGTGACATAATTGGACAAACTACCTACAGAGATTTAAAG
CTCTAAGGTAAATATAAAATTTTTAAGTGTATAATGTGTTAAACTACTGATTCTAATTGTTTGT
GTATTTTAGATTCCAACCTATGGAACTGATGAATGGGAGCAGTGGTGGAATGCCTTTAATGAGG
AAAACCTGTTTTGCTCAGAAGAAATGCCATCTAGTGATGATGAGGCTACTGCTGACTCTCAACA
TTCTACTCCTCCAAAAAAGAAGAGAAAGGTAGAAGACCCCAAGGACTTTCCTTCAGAATTGCTA
AGTTTTTTGAGTCATGCTGTGTTTAGTAATAGAACTCTTGCTTGCTTTGCTATTTACACCACAA
AGGAAAAAGCTGCACTGCTATACAAGAAAATTATGGAAAAATATTTGATGTATAGTGCCTTGAC
TAGAGATCATAATCAGCCATACCACATTTGTAGAGGTTTTACTTGCTTTAAAAAACCTCCCACA
CCTCCCCCTGAACCTGAAACATAAAATGAATGCAATTGTTGTTGTTAACTTGTTTATTGCAGCT
TATAATGGTTACAAATAAAGCAATAGCATCACAAATTTCACAAATAAAGCATTTTTTTCACTGC
ATTCTAGTTGTGGTTTGTCCAAACTCATCAATGTATCTTATCATGTCTGCATGCAGCTT**GGAG**
GC Homology arm2

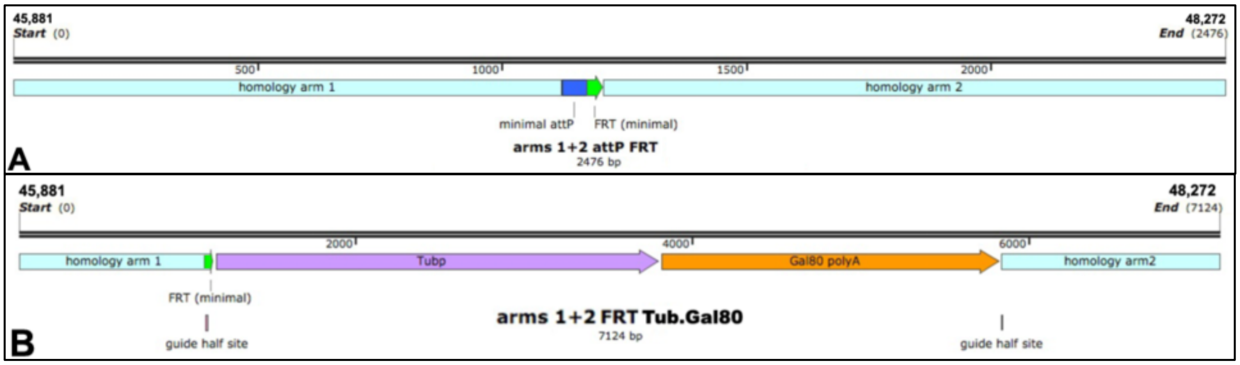

Fig. S4. FRT101F inserted transgenes after removal of DsRed. A) *TI{TI}FRT101F* with a single FRT. B) *TI{FRT.Tub.GAL80.O}101F* with distal Tub.GAL80. Below: the sequence of chromosome 4 from 45881bp to 48272bp is shown. The distal end of homology arm1 (blue) and the proximal end of homology arm2 (yellow) bracket FRT in both transgenes (red star).

45881bp begin Homology arm1
GCACGAGCTCTTGTGACATTGTCTACTCCGAAAAAGGAATGGTAATCTTTTAATTTTTTGATCC
TCTTCATTGTAATTAAAAATACGTCTCATTATGTAACACAGTTTTGGATTAAAAATCGAGCACA
CTACAAAATAAACTTTTAATAAAAAATATAAGACAACATAACATATATAAGTGAAAATAAAATG
TAAGAATCATGACGAAAAATGTGTTATTTTTATATTACACATAATTATATTTATTTATTATAGT
ACAATGACCAATATATTTACTAAAGGCAGCCATTATGAAGAATGCAAGGGGCAACATACATACA
TCAAAATTAAATCGAAAGAAGTTTATTTAAAAACTAAACAACACTCTGACCAAAAACCAATTAT
AATTAAAAAATCAATTAAAATATTAGCTAAGTGTGTGCTTTTTAAATACAAAATGAAACGTCTA
CTTGAGTTTTTAAACCTTTTATTCTTTTTCTAAATTCCTTAAAAAATATTTCAAAAAATGAAAT
TAAAATTTAAAATATTCAGGGGTTTCTTAAGGTAATTGAAACAGATCGAACTGCTATAAAATTG
TAATTTAAAATACATATATTTTATTAATCCATAATCCGAAATTTCGACCTCTTACTCCTACTGT
CTAAGTAAGAGATATCTGTTAGTCGAGGAAATAAGCTATAGCGTTCTGTCTTTGTAGGTTTCGG
TCAAAGACACTGGAATAACAAGACGCGTAACGGCCATACATTGGTTTCAAAATTGCGCTCTGTT
GTCCCCTCGCTTACGCTGAGAGCATAAGAAATCTAAAAATAGAATTTGCTTGCTTGTGTGAGTA
AAAACAATAGATATATGTATGTGTGTGTAGTTGTGCTAGAAGACGATTTTCGGGCCGAAATCAA
TTTTGTTTAAAGAAACAAGAAATGGATATTCGCCATTTTTGTATCGTATATGATGAAATAAAAT
AATAATTAAAAATTCACGCCGTGACTATTATAATTTTAAAGTTTTTTTATATTCGTTTGCTAAA
ATCGCCTCTTGAATTAGCTACCGTTTACATATTTATATTTATGTTAATAATTAATTATATGTAA
TATATGTAATAATCGGTACCCACGGGACAACACG*GGGAGGCCATTGCAATTGTAATTCACATG
TCGCAAGCAGAATACTCTAATGGCCTATATTCTAATATAAAGTCATTTTTAAAATTTATTTTGT
GATACTATGTACATAGATTCGGCAACTACTACTTTCAAGCTATATTTAAATAATAATAACATTA
AGGCAATGCAAATCAAGAATTTTTTACATGGTGCCAATTGATCAAACATAATATAGATTGAAAG
ATTAAGAACTTCTAAGATGAAGGGCAATACAATGCATGAGCATACGTGTGCACACATAAAGTTT
TCGGCTGCCACTGTATGCGTAGAAAAGAGCTGTTTGTTGTAGAGCTATCCGCTCTCTCGCTTTT
GAACAAAAATTCGTTAGAGCCTGGAGCCACATCTAGAGCCTCACCGAAAAAATCGTGCGCAAAA
AAATCTTATGACGTTACGCATCTCGTTATTGTAGTGTCTTTTTTTCGGTAAGGGATTGGAATGC
TTTTAGAATTTGAAATAACAAATATGTATGCCCCCTTACCGAATATATAAAACATTATTTTATA
ATTTTAATTATGCCATATAAATATAAGCCCACGAGTATGCAACGCCTTTTTTGTTAGTGGCGTC
ATAATTAATATTATTGTAATACCGGCTTACGCCGGTATTAAGGCTTATTAGGGCTTACGCCGGT
ATTAAGGGGAAAATAAATTAAAATAAATAAAAATAATTATTTAGTAAGAGCATAATCATTGCAT
TTTAATGTTTAAAATAGCTCGGAAGACATTATATTGTTACATGTATTTAGAATATTTAAAATAA
AATTCTCGTTTTTGTAAAATAATAAATGAACTTGATTAAAGGACATTTGTAAAAAATATTACAG
TGTTGAATCAGGATTTTAATTTAATTTTGTTTTGTAATTGGTATATTGCCTATAAAGTAAATAC
AATCTAAATAGAATAAAATAGTTTATTATGACTGATTTCTTGTCAGTGTTAGCTAGATAACATT
TTTTACTGCATCATTTGAAGGTATCTATTTTCCTCGAGTAAGGAAGTAAGGGATGTAGTCATAT
CGTTGATCACCATATTTGTCCCATGATATGGATGTGTAGAGCTGCTAACATCGGGAAACAATGT
ATTGTTTACCGGCATTCCCGGGAGGCTTTGTATTTGATTGGTAGATGTGTTGTTCGTCTCCATC
CAGTTTTGACAACTTTGTACATATTCCAGAGTACGCTGATATGTATCTAAATGCATAACCGAAG
TATTACTCGCTTGGGTGTCACAAAC **Homology arm2 end 48272bp**

II. Construction of *P{w[+mC]=dCORL.GAL4(-).PT}*

The genomic the region of the 4th encompassing *dCORL*, *sphinx*, and *toy* as well as the *dCORL* to *toy* intergenic segment contained in dCORL.GAL4 are indicated in Fig. 2A. The starting points for cloning the intergenic segment into a GAL4 expression vector were two subclones (*SmaI-PstI* and *XbaI-BamHI*; Fig. S5) from the 4th chromosome BAC BACR13D24 (GenBank AC010838) as reported in Takaesu et al. (2012).

Note that *dCORL* is transcribed anti-parallel to the direction of nucleotide numbering for the complete sequence (GenBank NC_004353). Sequence numbering is from proximal to distal while *dCORL* is transcribed from the opposite strand distal to proximal. *dCORL* transcription is also in the opposite direction from *sphinx* and *toy* transcription (Fig. S5).

Our experience with the complicated array of tissue- and stage-specific intertwined enhancers and suppressors in the *dCORL* to *toy* intergenic region (Tran et al. 2018a) led us to clone the intergenic region in both orientations upstream of GAL4. We named the transgene oriented in the direction of *dCORL* transcription (i.e., with GAL4 in the location of *dCORL* in the genome) the forward (+) orientation. We named the transgene oriented in the direction of *toy* transcription and that matches the proximal to distal orientation sequence numbering (i.e., with GAL4 in the location of *toy* in the genome) the reverse (-) orientation.

Reverse orientation cloning strategy for dCORL.GAL4(-): The goal was to subclone the *dCORL* to *toy* intergenic region into the pPT.GAL4 vector (Sharma et al. 2002) in *BamHI-SmaI* orientation with *BamHI* from *toy* intron1 adjacent to GAL4. In this transgene *toy* exon1 and GAL4 are transcribed in the same direction and this reflects the orientation of numbering in the chromosome (Fig. S5).

This was done with NEBuilder (New England Biolabs; Ipswich, MA).

1. To create pPT.GAL4.dCORL.*BamHI-BstEII*: a) digested pPT.GAL4 with *StuI* and *NotI*; b) PCR of *dCORL* fragment *BamHI-BstEII* from parent *XbaI-BamHI* clone; c) subclone PCR product into digested vector by using NEBuilder; d) sequence and midiprep.

2. To create pPT.GAL4.dCORL.*BamHI-XbaI*: a) digested above pPT.GAL4.dCORL.*BamHI-BstEII* with *BstEII*; b) PCR of *dCORL* fragment *BstEII-XbaI* from parent *XbaI-BamHI* clone; c) subclone PCR product into digested vector via NEBuilder; d) sequence and midiprep.

3. To create pPT.GAL4.dCORL.*BamHI-SmaI*: a) digested above pPT.GAL4.dCORL.*BamHI-XbaI* with *XbaI*; b) PCR of *dCORL* fragment *XbaI-SmaI* from parent *SmaI-PstI*; c) subclone PCR product into digested vector via NEBuilder; d) sequence and midiprep.

Forward orientation cloning strategy for dCORL.GAL4(+): The goal was to subclone the *dCORL* to *toy* intergenic region into the pPT.GAL4 vector (Sharma et al. 2002) in *SmaI-BamHI* orientation with *SmaI* from *dCORL* intron1 adjacent to GAL4. In this transgene *dCORL* exon1 and GAL4 are transcribed in the same direction with the noncoding RNA *sphinx* divergently transcribed between them and this is antiparallel to the orientation of numbering in the chromosome (Fig. S6).

The initial subcloning step placed the *dCORL* to *toy* intergenic region fragment *XbaI-BamHI* into the pPT.GAL4 vector utilizing restriction enzymes. The second step placed the remaining *SmaI–XbaI* fragment into the above pPT.GAL4.dCORL.*XbaI-BamHI* clone utilizing NEBuilder.

1. To create pPT.GAL4.dCORL.*XbaI-BamHI*: a) digested both the *XbaI-BamHI* parent clone and pPT.GAL4 with *BamHI* and *XbaI*; b) ligated the *dCORL XbaI-BamHI* fragment into digested pPT.GAL4 vector; c) sequence and midiprep.

2. To create pPT.GAL4.dCORL.*SmaI-BamHI*: a) digested pPT.GAL4.dCORL.*XbaI-BamHI* with *XbaI*; b) PCR of *dCORL* fragment *SmaI-XbaI* from parent *SmaI-PstI* clone; c) subclone PCR product into digested vector via NEBuilder; d) sequence and midiprep.

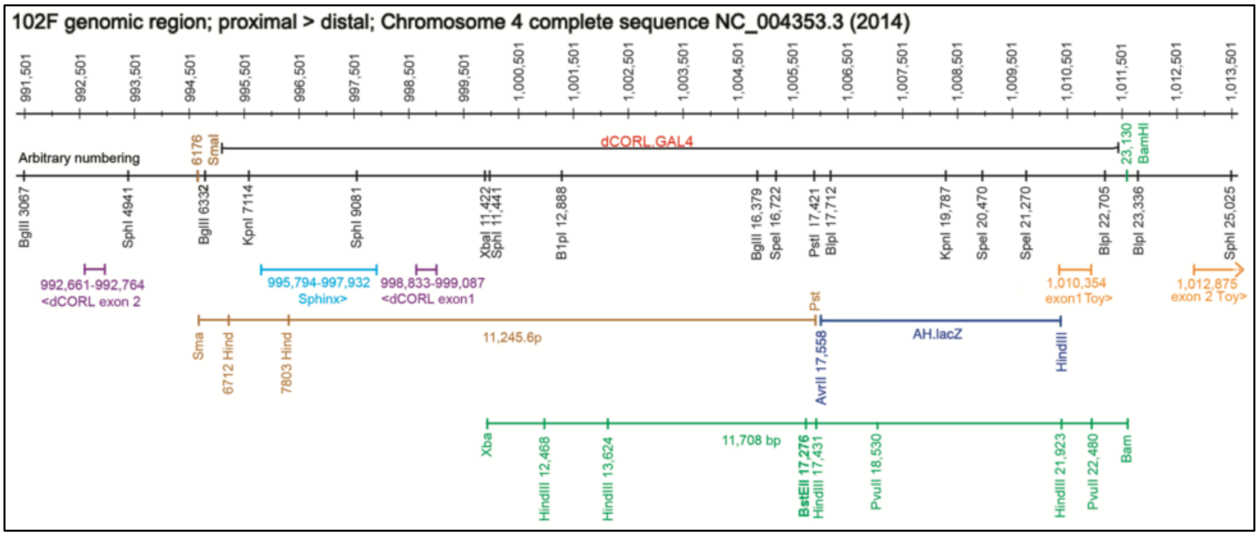

Fig. S5. *dCORL* genomic map and dCORL.GAL4 parental clones. Roughly 20kb within chromosome band 102F on the 4th chromosome is shown. The coordinate line represents nucleotide numbering from the Release 5.0 of the *Drosophila melanogaster* genome (4th chromosome; NC_004353.3; 2014). A second coordinate line reflects the same region with restriction sites given arbitrary numbers based on their position in the 4th chromosome BAC BACR13D24 (GenBank AC010838). Between the two coordinate lines the segment corresponding to dCORL.GAL4 is shown. Below the coordinate lines the relevant portions of the intron-exon structure of *dCORL*, *sphinx* and *toy* are shown. Each gene is a distinct color and their direction of transcription is shown with an arrowhead. The bottom brown and green lines are the parental subclones of dCORL.GAL4 (*SmaI* to *PstI* and *XbaI* to *BamHI*). The dark blue line represents the well characterized dCORL reporter gene - AH.lacZ. This reporter is expressed in dILP2 expressing neurons but does not reflect *toy* expression (Tran et al. 2018b).

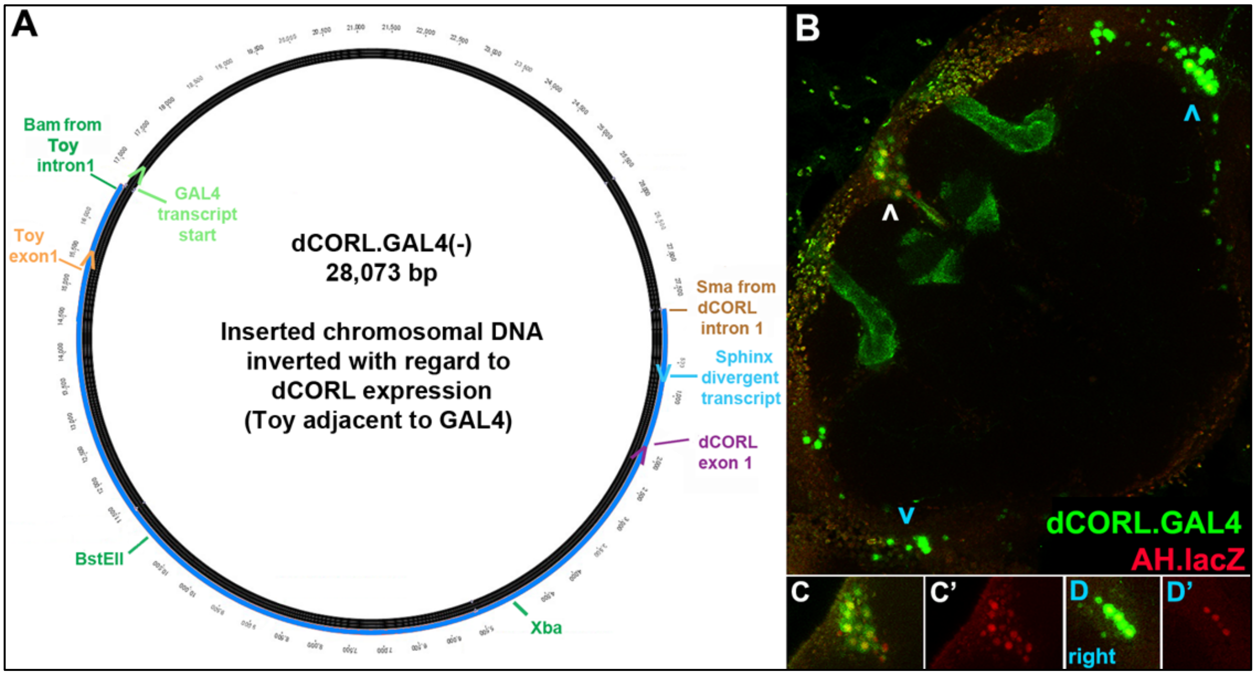

Fig. S6. ***P{w[+mC]=dCORL.GAL4(-).PT}* transgene map.** A) The complete plasmid with the *dCORL* to *toy* intergenic region (blue highlight) cloned into pPT.GAL is shown. The relevant portions of the intron-exon structure of *dCORL*, *sphinx* and *toy* are noted. Each gene is a distinct color and their direction of transcription is shown with an arrowhead. Restrictions enzyme sites relevant to the cloning process are shown. In this orientation *toy* exon1 is adjacent to GAL4 and transcribed in the same direction (arrowheads). Notwithstanding the fact that *toy* exon1 and GAL4 are adjacent with *dCORL* divergently transcribed from the opposite end, this orientation drove GAL4 in a phenocopy of *dCORL* expression. This transgene was recombined onto a 2nd chromosome carrying UAS.GFP. We refer to the recombinant chromosome as dCORL.GAL4. B) One day old adult female brains reflecting dCORL.GAL4 (green) and the well characterized dCORL reporter AH.lacZ (red; Tran et al. 2018a,b). Small stack showing GFP and lacZ in three regions: mushroom body, IPC neurons (white arrowhead), and the lobula plate (blue arrowheads). C,C') Small stack in two colors or red (lacZ) alone indicating that not all IPC neurons that express lacZ express dCORL.GAL4. D,D') Single slice in two colors or red (lacZ) alone indicating that four lobula plate neurons express both lacZ and dCORL.GAL4 validating them as *dCORL* expressing cells.

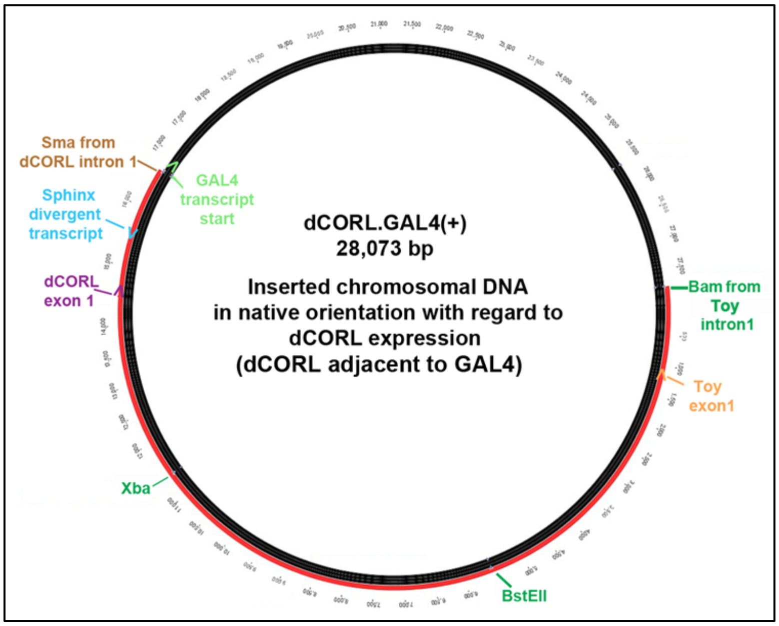

Fig. S7. ***P{w[+mC]=dCORL.GAL4(+).PT}* transgene map.** The complete plasmid as shown above with the *dCORL* to *toy* intergenic region (red highlight) cloned into pPT.GAL. In this orientation *dCORL* exon1 is adjacent to GAL4 and transcribed in the same direction (arrowheads). Multiple transgenic lines were obtained, crossed to UAS.GFP and one day old adult female progeny examined for similarity to *dCORL* RNA in situ expression. No expression was seen in any adult brains indicating that repressors in the region overrule intertwined enhancers in this orientation.

III. *sphinx* RNA in situ.

sphinx genomic DNA from 976,042bp to 976,512bp was employed as a probe (Fig. S8). This region was chosen because it does not fall within any exons of *dCORL*. It is located upstream of *dCORL* transcript -RC, within the first intron of transcript-RB and within the second intron of transcript -RD. The region also contains exonic sequence from two *sphinx* transcripts -RB and -RC, although it is in intronic for transcript-RA. A previous report noted that unspliced *sphinx* transcripts were viable in larvae (Wang et al. 2002) suggesting that this probe will detect all transcripts of *sphinx* and none of *dCORL*.

sphinx genomic DNA was amplified via primers *sphinx* 03F 5' GAGATCGGTTCTTCTCGATAC3' and *sphinx* 04R 5'CGGTATACCAAAAACACTGTGG3'. The product was cloned with the TOPO TA Cloning Kit (Invitrogen). Orientation was verified by sequencing with the T7 primer, and both orientations saved. The "sense" clone is transcribed by T7 polymerase in the direction of *sphinx* transcription and the "anti-sense" clone is transcribed in the direction opposite to *sphinx* transcription. Clones were linearized with *BamHI*.

For probe preparation the method of Kosman et al. (2004) was utilized with the following modifications: DIG RNA Labeling Mix (Roche) was employed, treatment with 1μL of DNaseI (1 unit/μL) at 37° for 15 minutes removed template DNA and hydrolysis to small fragments via incubation at 60° for 60 minutes. Probes were resuspended in hybridization solution with 0.2% TritonX-100 at 10-20 ng/μL. Third instar larval brains were dissected, fixed, transferred to PBT with 0.2% TritonX-100 and treated with 5 μg/mL proteinaseK in PBT for 5 minutes at room temperature. Hybridization at 55° for 20 hours was performed in a small volume (30 μL) with 4 μL of probe (final concentration 1.3-2.6 ng/μL). After extensive washing, Anti-Digoxigenin-AP Fab fragments (Roche) were added, followed by AP colorimetric development with nitroblue tetrazolium and 5-bromo-4-chloro-3-indolyl phosphate for 1 hour. Larval brains were mounted in 80% glycerol/20% PBTriton (0.2%) for photography.

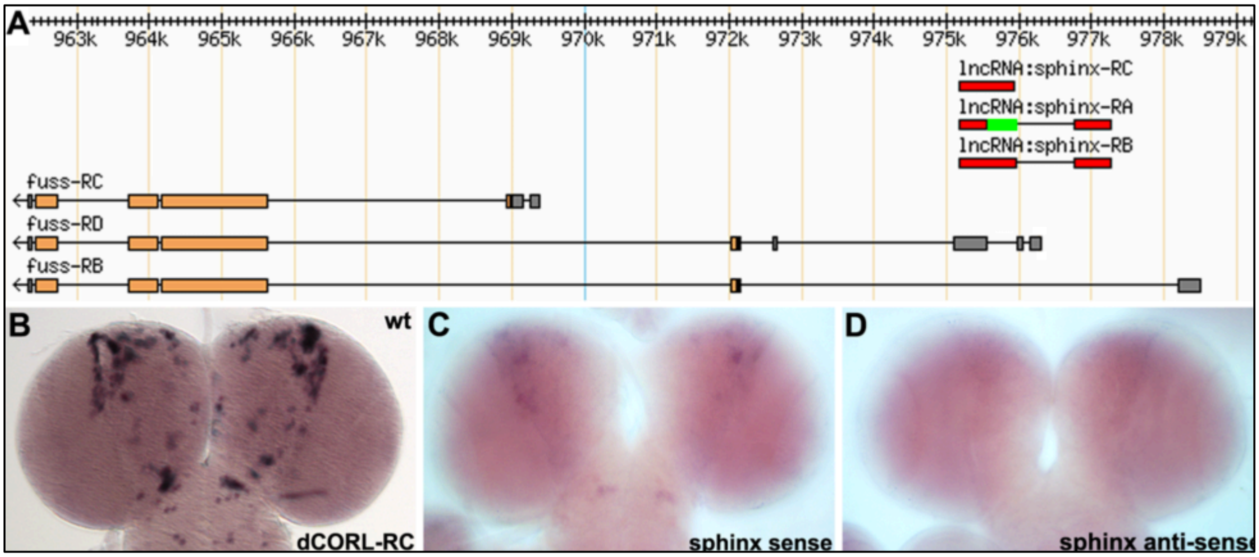

Fig. S8. *sphinx* RNA in situ shows no larval brain expression. A) Flybase Gbrowse image of the genomic region revealing the exonic structure of *sphinx* and *dCORL* (*fuss* in Flybase). Orange boxes indicate protein coding exons in *dCORL* and red boxes non-coding RNA exons in *sphinx*. The segment indicated in green (sequence below) was chosen for a *sphinx* probe because it is predicted to detect multiple transcripts of *sphinx* and none of *dCORL*. B) *dCORL* larval brain RNA in situ employing a probe from dCORL transcript-RC composed primarily of the ORF exons common to all transcripts. Strong expression is seen (Takaesu et al. 2012). C) Sense strand of the *sphinx* probe is antisense for *dCORL*. A low level of unspliced *dCORL* transcripts RB and RD are detected. D) The antisense strand of the *sphinx* probe. No *sphinx* expression is detected. Non-specific expression at the medulla/lobula border is seen with both strands.

sphinx is transcribed in parallel to chromosome 4 numbering and the probe sequence (976,042 - 976,512bp) is shown.

```
5’GAGATCGGTTCTTCTCGATACCGACTTCTTCTGGACATCGAGAAGACATTCTCATTTCGATA
GCTATTTGCACACTGAAAATGTTTCGATCTCGATATGCGTATGCTGAGTACGATTTCATCTTGT
CCAAATTTTAGATCGAAATGAGTATATGAGTAACGGCAATAGCAATATTTCGTCACCCGTTGTT
TAAAAACAATACAAGAAATAAAAAACTCAGCAAAAAAATACGTTTTTATCACGAATTTTTTTTA
ATGCTGTAGAGCCCTTGTAATTCCCTAACATCGCAGGTTTAAAAGTTTAAGCAACATTAAACAG
CCCCTTTGCAAATATGAAAGAACTAACCCTTTTTATAACAAAAAAATATTTCGTCATATAAATT
ATAAAAATTGTTTAGAAGTGTGGTCGTGAAAATTTTGGGAGTTTTGTAGGTCAAAGGAGGGGCG
TGGCCACAGTGTTTTTGGTATACCG 3’
```
